# Multi-omics gut microbiome signatures in obese women: role of diet and uncontrolled eating behavior

**DOI:** 10.1186/s12916-022-02689-3

**Published:** 2022-12-27

**Authors:** Monica Barone, Silvia Garelli, Simone Rampelli, Alessandro Agostini, Silke Matysik, Federica D’Amico, Sabrina Krautbauer, Roberta Mazza, Nicola Salituro, Flaminia Fanelli, Patricia Iozzo, Yolanda Sanz, Marco Candela, Patrizia Brigidi, Uberto Pagotto, Silvia Turroni

**Affiliations:** 1 Microbiomics Unit, Department of Medical and Surgical Sciences, University of Bologna, 40138 Bologna, Italy; 2 Unit of Microbiome Science and Biotechnology, Department of Pharmacy and Biotechnology, University of Bologna, 40126 Bologna, Italy; 3 Unit of Endocrinology and Prevention and Care of Diabetes, Center for Applied Biomedical Research, S. Orsola Polyclinic, Istituto Di Ricovero E Cure a Carattere Scientifico (IRCCS), Department of Medical and Surgical Sciences, University of Bologna, 40138 Bologna, Italy; 4 Department of Experimental, Diagnostic, and Specialty Medicine, S. Orsola Polyclinic, Istituto Di Ricovero E Cure a Carattere Scientifico (IRCCS), University of Bologna, 40138 Bologna, Italy; 5 Institute of Clinical Chemistry and Laboratory Medicine, University Hospital Regensburg, 93053 Regensburg, Germany; 6 Present Address: Research Development - Life Sciences and Bioeconomy Unit, Research Services Division (ARIC), University of Bologna, 40126 Bologna, Italy; 7 Institute of Clinical Physiology, National Research Council, 56124 Pisa, Italy; 8 Microbial Ecology, Nutrition & Health Research Unit, Institute of Agrochemistry and Food Technology, Spanish National Research Council (IATA-CSIC), 46980 Valencia, Spain

**Keywords:** Gut microbiome, Obesity, Diet, Uncontrolled eating behavior, Metagenomics, Metatranscriptomics, Lipidomics, Gut-brain axis

## Abstract

**Background:**

Obesity and related co-morbidities represent a major health challenge nowadays, with a rapidly increasing incidence worldwide. The gut microbiome has recently emerged as a key modifier of human health that can affect the development and progression of obesity, largely due to its involvement in the regulation of food intake and metabolism. However, there are still few studies that have in-depth explored the functionality of the human gut microbiome in obesity and even fewer that have examined its relationship to eating behaviors.

**Methods:**

In an attempt to advance our knowledge of the gut-microbiome-brain axis in the obese phenotype, we thoroughly characterized the gut microbiome signatures of obesity in a well-phenotyped Italian female cohort from the NeuroFAST and MyNewGut EU FP7 projects. Fecal samples were collected from 63 overweight/obese and 37 normal-weight women and analyzed via a multi-omics approach combining 16S rRNA amplicon sequencing, metagenomics, metatranscriptomics, and lipidomics. Associations with anthropometric, clinical, biochemical, and nutritional data were then sought, with particular attention to cognitive and behavioral domains of eating.

**Results:**

We identified four compositional clusters of the gut microbiome in our cohort that, although not distinctly associated with weight status, correlated differently with eating habits and behaviors. These clusters also differed in functional features, i.e., transcriptional activity and fecal metabolites. In particular, obese women with uncontrolled eating behavior were mostly characterized by low-diversity microbial steady states, with few and poorly interconnected species (e.g., *Ruminococcus torques* and *Bifidobacterium* spp.), which exhibited low transcriptional activity, especially of genes involved in secondary bile acid biosynthesis and neuroendocrine signaling (i.e., production of neurotransmitters, indoles and ligands for cannabinoid receptors). Consistently, high amounts of primary bile acids as well as sterols were found in their feces.

**Conclusions:**

By finding peculiar gut microbiome profiles associated with eating patterns, we laid the foundation for elucidating gut-brain axis communication in the obese phenotype. Subject to confirmation of the hypotheses herein generated, our work could help guide the design of microbiome-based precision interventions, aimed at rewiring microbial networks to support a healthy diet-microbiome-gut-brain axis, thus counteracting obesity and related complications.

**Supplementary Information:**

The online version contains supplementary material available at 10.1186/s12916-022-02689-3.

## Background

Over the last 4 decades, the worldwide prevalence of overweight and obesity has risen from 3.2 to 10.8% in men and from 6.4 to 14.9% in women [1]. In Italy, the predicted 2025 prevalence of obesity in adults has been estimated at 25.5% in men and 22.9% in women [2]. Obesity is recognized as a complex, multifactorial disease that represents a major risk factor for health, with important consequences on quality of life, life expectancy and healthcare costs [3]. In particular, obesity has been linked to increased risk of developing a wide range of non-communicable diseases, such as type 2 diabetes, fatty liver disease, hypertension, dyslipidemia, coronary heart disease, stroke, and cancer [3]. Despite steady progress in the management of obesity and its comorbidities, preventive and therapeutic strategies sometimes prove ineffective, and the long-term maintenance of weight loss is particularly challenging. In order to design tailored treatment strategies that are effective over time, the need to better stratify patients according to precise phenotyping criteria has recently been highlighted [4]. To this end, a systems biology-oriented approach that elucidates the different components of the obesity phenotype and their interactions is strongly advocated, for practical advancement in obesity research towards precision and personalized medicine [3, 5].

In this scenario, increasing efforts have been made to investigate the relationship between obesity and the gut microbiome (GM), which has led to the identification of the latter as a potential viable biomarker and therapeutic target [6, 7]. Composed mainly of bacteria, along with archaea, fungi, and viruses, the trillion-member community that resides in the human gastrointestinal tract has emerged as a key regulator of host metabolism, playing a crucial role in the pathophysiology of obesity by contributing to increased energy harvesting and storage, affecting adipose tissue composition and fat mass gain, as well as providing low-grade inflammation and insulin resistance [6]. These actions are mediated by a plethora of bioactive metabolites produced by the GM in a diet-dependent manner, e.g., short-chain fatty acids (SCFAs), conjugated fatty acids, and tryptophan metabolites. These molecules can in fact also exert peripheral effects and even modulate the brain, through direct or indirect mechanisms involving a complex network of neuroendocrine factors and their receptors, thus affecting central appetite control, including food reward signaling, and energy balance [8, 9]. It is therefore not surprising that perturbations of the gut-microbiome-brain axis have been implicated in unbalanced eating patterns towards cravings, overeating, and hedonic-driven eating behavior [10]. In particular, “food addiction” (FA), a type of eating behavior in which the hedonic aspect overrides energy homeostatic mechanisms, has been recognized to play a crucial role in the pathophysiology of obesity, especially in women [11–13]. Despite the ongoing controversy over its diagnosis [14–16], addictive eating behavior leads to the increased consumption of highly palatable foods well beyond the energy needs and despite the known negative physical, physiological, and psychological consequences [11, 17]. Recently, FA in obese females has been associated with GM dysbiosis and reduced levels of indolepropionate, a neuroprotective tryptophan-derived microbial metabolite, which probably exerts both local effects by strengthening barrier function, and peripheral effects, e.g., preserving beta cell function and counteracting oxidative stress and inflammation of the central nervous system [18]. Very recently, Leyrolle and colleagues have examined the biological and psychiatric profile of obese patients with and without binge eating disorders through non-targeted multi-omics approaches. The authors suggest the potential role of certain GM layouts (i.e., with enrichment of *Bifidobacterium* and *Anaerostipes*, and depletion of *Akkermansia* and *Intestimonas*) and/or plasma metabolites (i.e., food contaminants and food derived-metabolites) as drivers or biomarkers of binge eating disorders [19]. However, to our knowledge, no other studies have examined the relationship between GM and FA and, in general, very little information is available on GM functionality in uncontrolled eating behavior and obesity.

In an attempt to bridge this gap, providing some insight into the complex gut-microbiome-brain axis in obesity, here we applied an exploratory multi-omics approach to a cohort of 37 normal-weight and 63 overweight/obese premenopausal women, enrolled within the European FP7 NeuroFAST and MyNewGut projects, with different eating habits and behaviors. Specifically, we first characterized the compositional profiles of GM by 16S rRNA amplicon sequencing and shotgun metagenomics, for fine taxonomic resolution down to species level, and evaluated their association with eating behavior as assessed by several psychometric questionnaires. We then applied a shotgun metatranscriptomic approach to unravel transcriptionally active microbial pathways associated with eating behavior, as well as variations in specific microbial genes involved in the regulation of food intake, energy expenditure, and neuroendocrine signaling. Metabolic outputs of the GM, such as SCFAs, bile acids, and sterols, were finally assessed through fecal lipidomics. Our findings, albeit exploratory and associative, provide potential GM-based biomarkers to phenotype the patient with excess weight and FA/nutritional dysfunction to personalize future treatments.

## Methods

### The NeuroFAST cohort: study design and sample collection

The present study is based on a subgroup of 63 overweight/obese (OB) women enrolled in the project NeuroFAST (fully described in a previous publication) [20]. Study population included women aged > 18 years in a premenopausal state, with BMI ranging between 24.9 and 40.0 kg·m^−2^. Overweight and obesity were defined according to the World Health Organization criteria [21]. Exclusion criteria were the presence of acute/chronic diseases (i.e., type 2 diabetes, thyroid dysfunction, endogenous hypercortisolism or other endocrine or metabolic disorders, major cardiovascular events, renal, hepatic, and systemic diseases, central nervous system illness and cancer), previous and current neurological or psychiatric disease (explored by a direct psychological interview, the Mini-International Neuropsychiatric Interview—MINI [22]), and current use of psychotropic medication, corticosteroid therapy, post-menopausal state, pregnancy, or nursing. Additional exclusion criteria were as follows: alcohol and substance abuse and addiction, anorexia nervosa, bulimia nervosa, ongoing or recent (i.e., the last 6 months) diet, and treatment with any medication in the past 6 months before clinical examination. The study cohort was implemented in the context of the project MyNewGut, by enrolling 37 healthy normal-weight (NW) women. The abovementioned exclusion criteria were maintained. Both OB and NW women were enrolled at the Unit of Endocrinology and Prevention and Care of Diabetes of S. Orsola Polyclinic—University Hospital (Bologna, Italy). In order to exclude country-related effects on the GM profile, all women were enrolled in the Emilia Romagna region and surroundings (Italy). Psychometric and nutritional questionnaires were administered as described below. Stool and blood were collected from all women for GM analysis and measurement of biochemical profile, metabolic and inflammatory biomarkers, and gut hormones, respectively. Moreover, all participants underwent an oral glucose tolerance test. Venous blood was drawn in the follicular phase of the ovarian cycle after an overnight fast using standardized procedures, to minimize the effect of hormonal fluctuations on the experimental results [23]. Routine biochemical parameters, serum hormones, and metabolites were measured at the Central Laboratory of S. Orsola Polyclinic University Hospital (Bologna, Italy); additional blood samples for gut hormones were collected and stored at – 80 °C up to the assay. Fecal samples were collected within 24–48 h prior to clinical and nutritional assessment, stored at – 20 °C on the day of collection and then transferred to – 80 °C upon arrival in the laboratory. The study was conducted in accordance with the Declaration of Helsinki and approved by the local ethics committee (072/2010/U/Sper; 149/2015/U/Sper). A written informed consent was provided by each woman enrolled.

### Collection of clinical, behavioral, and nutritional data

Examinations of the enrolled participants included anthropometric data (i.e., body weight, height, BMI, waist and hip circumferences, and waist-to-hip ratio), systolic and diastolic blood pressure, and physiological markers in blood. Insulin resistance and sensitivity were defined according to the Homeostasis Model Assessment of Insulin Resistance (HOMA-IR) [24] and the Matsuda-index [25] respectively. A HOMA-IR value > 2.5 was used to define insulin-resistant participants. All women underwent a visit with a fully trained psychologist from the Department of Experimental, Diagnostic and Specialty Medicine (University of Bologna), aimed at investigating the previous or current presence of psychopathological disorders, and use of psychotropic agents, through MINI [22]. In addition, participants filled out a battery of psychometric questionnaires:

- Bulimic Investigatory Test Edinburgh (BITE): a 33-item self-report measure designed to assess symptomology of bulimia or binge eating, consisting of two subscales: the Symptom Scale, measuring the degree of symptoms, and the Severity Scale, providing an index of the severity of symptoms [26]. A Symptom Scale score of 20 or more indicates the presence of binge eating symptoms; a Severity Scale score of 10 or more indicates a high degree of severity. High scores on both scales indicate a high probability that a participant will fulfill the criteria for an eating disorder diagnosis. BITE is considered a valid and reliable questionnaire [27] and is widely used to assess binge eating symptomology and to screen for bulimic symptoms and their severity;
- Three-factor eating questionnaire (TFEQ): a self-report questionnaire, containing 18 items, widely used in eating behavior research in both OB and NW subjects [28]. Participants must rate each item using a 4-point Likert scale in which 1 is definitively true and 4 is definitively false. TFEQ was designed to assess three cognitive and behavioral domains of eating (factors): cognitive restraint (CR), uncontrolled eating (UE), and emotional eating (EE). The CR scale contains six items and refers to the conscious restriction of food intake to control body weight or promote weight loss. The UE scale contains nine items and refers to the tendency to eat more than usual because of a loss of control over intake. The EE scale contains three items and refers to overeating during dysphoric mood state. The TFEQ-18R was derived from a previous 51-item and 21-item version. The current version has shown a robust factor structure, good validity and internal reliability, and no significant ceiling or floor effects [29];
- Yale Food Addiction Scale (YFAS): a questionnaire developed by Gearhardt and colleagues to operationalize FA, including 25 items to assess signs of substance-dependence symptoms (e.g., tolerance, withdrawal, loss of control) in eating behavior over the past 12 months [11]. YFAS provides two scoring options: a symptom count version and a diagnostic version. To receive a diagnosis of FA, it is necessary to report experiencing three or more symptoms and clinically significant impairment or distress. Moreover, based on symptoms count, Gearhardt and colleagues suggested to dichotomize participants in high and low food addicted groups [12]. Consequently, participants with 3 or more symptoms were considered as highly food addicted while low food addicted participants scored two or fewer symptoms [23]. Accordingly, for the present study, OB participants were stratified into three groups: high addictive eating behavior, including participants with three or more symptoms and FA diagnosis (O_DHA); high addictive eating behavior, including participants with three or more symptoms but no FA diagnosis (O_HA); low addictive eating behavior, including participants with two or less symptoms (O_LA). The recently released YFAS 2.0 version [30] was not used in the present study because it was not available at the time of recruitment;
- Perceived Stress Scale (PSS): a 10-item, self-report questionnaire, assessing the individual experience of perceived stress over the past month [31, 32]. Participants must rate the frequency with which they have perceived situations as stressful using a 5-point Likert scale in which 0 is never and 4 is very often. Scores ranging from 27 to 40 are considered high perceived stress. PSS has been shown to be a reliable and valid measure of psychological stress.

Nutritional questionnaires were also administered to obtain information on the frequency of consumption (no. of portions per month) of every category of food (Food Frequency Questionnaire, FFQ) [33]. Fiber intake was normalized to 1000 kcal, following the same approach as Rampelli et al. [7]. Information about personal/familiar anamnesis, menstrual history and pregnancies, body weight curve (recall of body weight values since the age of 18 to the present, in order to identify the occurrence of “stress-related weight gain”), and prior and current medications was also collected.

### Microbial DNA extraction

Total microbial DNA was extracted from fecal samples by the repeated bead-beating plus column method [34], with only slight modifications as reported by Barone et al. [35]. Briefly, 250 mg of fecal samples were suspended in 1 ml of lysis buffer (500 mM NaCl, 50 mM Tris–HCl pH 8, 50 mM EDTA, 4% (w/v) SDS), added with four 3-mm glass beads and 0.5 g of 0.1-mm zirconia beads (BioSpec Products) and homogenized using a FastPrep instrument (MP Biomedicals) with three bead-beating steps at 5.5 movements/sec for 1 min, and 5-min incubation in ice between treatments. After incubation at 95 °C for 15 min, stool particles were pelleted by centrifugation at 14,000 rpm for 5 min. Nucleic acids were precipitated by adding 260 μl of 10 M ammonium acetate and one volume of isopropanol. The pellets were then washed with 70% ethanol and suspended in TE buffer. RNA was removed by treatment with 2 μl of DNase-free Rnase (10 mg/ml) at 37 °C for 15 min. Protein removal and column-based DNA purification were performed following the manufacturer’s instructions (QIAGEN). DNA was quantified with the NanoDrop ND-1000 spectrophotometer (NanoDrop Technologies).

### 16S rRNA gene sequencing and bioinformatics

The V3-V4 hypervariable regions of the 16S rRNA gene were amplified using the 341F and 805R primers with added Illumina adapter overhang sequences as previously reported by Barone et al. [35]. PCR products of around 460 bp were purified using a magnetic bead-based system (Agencourt AMPure XP; Beckman Coulter). Each indexed library was prepared by limited-cycle PCR using Nextera technology and further purified as described above. The libraries were subsequently pooled at equimolar concentration, denatured with 0.2 N NaOH, and diluted to 6 pM with 20% PhiX control. Sequencing was performed on an Illumina MiSeq platform using a 2 × 250 bp paired-end protocol, according to the manufacturer’s instructions (Illumina). Raw sequence data are available for download from the NCBI Sequence Read Archive (BioProject ID: PRJNA832282).

Paired-end reads were processed combining PANDAseq [36] and QIIME [37]. High-quality sequences were clustered into OTUs at 97% sequence similarity by UCLUST [38] and taxonomy was assigned against the Greengenes database (May 2013 release). All singleton OTUs were discarded. Alpha diversity was evaluated using two different metrics: Shannon index and number of observed OTUs. Beta diversity was estimated by computing weighted and unweighted UniFrac distances, which were used as input for principal coordinates analysis (PCoA). PCoA, heatmap, and bar plots were built using the R packages made4 [39] and vegan (http://www.cran.r-project.org/package=vegan). The obtained OTUs were filtered for a prevalence in participants of at least 20%, and hierarchical Ward linkage clustering based on Spearman correlation coefficients of the proportion of OTUs was used to identify microbiome steady states. Multiple testing using the Benjamini–Hochberg method allowed verifying that each cluster showed significant Spearman correlations between samples within the group. Significant differences between clusters were evaluated with permutational MANOVA using the Spearman distance matrix as input (function adonis of the vegan package in R).

### Co-abundance analysis

Co-abundance groups (CAGs) were identified as previously described [40]. Briefly, the dataset included the bacterial genera present in at least two samples with relative abundance > 0.1%. Kendall’s correlation test was used to evaluate associations between bacterial genera. The identified associations were visualized using hierarchical Ward linkage clustering with distance metrics based on Spearman’s correlation and used to determine the co-abundance of bacterial groups. Significant associations were checked for multiple tests using the *q*-value method (false discovery rate, FDR ≤ 0.05) [41] and plotted within the networks. Permutational MANOVA using the Kendall distance matrix as input was applied to assess whether the CAGs were significantly different from each other. Cytoscape software was used to create Wiggum plot networks (http://www.cytoscape.org), as previously reported [40]. In such graphs, the size of the circle represents the bacterial abundance, while the connections between the nodes represent positive and significant Kendall correlations between bacterial genera (FDR ≤ 0.05).

### Shotgun metagenomic DNA sequencing and data analysis

Metagenomic DNA libraries were prepared using the QIAseq FX DNA Library Kit, following the manufacturer’s instructions (QIAGEN). Briefly, for each sample, 100 ng of DNA were fragmented to 450-bp size, end-repaired, and A-tailed using FX Enzyme Mix with the following thermal cycle: 4 °C for 1 min, 32 °C for 8 min, and 65 °C for 30 min. Samples were then incubated at 20 °C for 15 min in the presence of DNA ligase and Illumina adapter barcodes for indexing and adapter ligation. After two purification steps with Agencourt AMPure XP magnetic beads (Beckman Coulter), 10-cycle PCR amplification, and a further step of purification as above, samples were pooled at equimolar concentration of 4 nM. Sequencing was performed on an Illumina NextSeq 500 platform using a 2 × 150 bp paired-end protocol, following the manufacturer’s instructions (Illumina). Raw sequence data are available for download from the NCBI Sequence Read Archive (BioProject ID: PRJNA832560). Species-level characterization of shotgun metagenomics data was conducted as previously described by Rampelli and colleagues [42]. In brief, shotgun reads were first filtered by quality and human sequences by means of the human sequence removal pipeline and the WGS read processing procedure of the HMP Consortium [43]. The obtained reads were taxonomically characterized at species level by MetaPhlAn2 [44].

### RNA isolation, sequencing, and bioinformatics

RNA extraction was carried out using the RNeasy PowerMicrobiome kit (QIAGEN), according to the manufacturer’s instructions. In brief, 250 mg of stool samples were processed by adding the chemical lysis buffer (PM1/β-mercaptoethanol) and subsequent homogenization using a FastPrep instrument (MP Biomedicals) at 5.5 movements/sec for 1 min. DNA was removed by on-column DNase treatment, followed by a washing step to remove the enzyme and any digested nucleic acids. The purified RNA was eluted in RNase-free water. For each sample, rRNA was depleted using the Ribo-Zero Gold kit for bacteria (Illumina) according to the manufacturer’s instructions. In short, total RNA was hybridized with rRNA Removal Beads (Illumina) and a subsequent clean-up was performed using the RNAClean XP beads (Illumina) following the RNA denaturation step. RNA libraries were prepared using the TruSeq Stranded Total RNA library kit (Illumina), according to the manufacturer’s instructions. In brief, after reverse transcription, the synthesis of the second strand was performed with a combination of enzymes and optimized buffer solutions to allow the degradation of the RNA strand, the generation of a second cDNA strand, and the generation of blunt DNA ends. Subsequent addition of the A-base ensured efficient ligation of Illumina-compatible adapters. The generated RNA-seq libraries were PCR-amplified, purified with magnetic bead-based clean-ups (Agencourt AMPure XP; Beckman Coulter), and pooled at equimolar concentration of 4 nM before being loaded onto the flow cell. Sequencing was performed on an Illumina NextSeq 500 platform using a 2 × 150 bp paired-end protocol, following the manufacturer’s instructions (Illumina). Raw sequence data are available for download from the NCBI Sequence Read Archive (BioProject ID: PRJNA832581).

Metatranscriptomic reads passed through the same pipeline used in the metagenomic dataset in order to remove low-quality bases, reads of human origin, and reads encoding for rRNA. Metatranscriptomes were functionally profiled using HUMAnN2 [45] to quantify expression levels of genes and pathways. Reads were aligned to sample-specific pangenomes, i.e., all gene families in any microorganism detected in a given sample, using Bowtie and the UniRef90, MinPath, and KEGG databases [46–49]. Hits were counted per KEGG pathway and normalized for length, alignment quality score, and sequencing depth. HUMAnN2 RNA-level outputs (transcript abundances) were then normalized by the corresponding DNA-level outputs from metagenomic results to quantify microbial expression regardless of gene copy number. To this aim, the shotgun metagenomics data were analyzed with HUMAnN2 using the same parameters reported above for the metatranscriptomic data.

### Lipidomics analysis

The targeted lipidomics analysis included in our work was originally conducted by Matysik and colleagues [50]. Briefly, 2.5 ml of 70% isopropanol was added to each stool sample and homogenized in a gentleMACS dissociator (Miltenyi Biotec GmbH). Samples were kept on ice between each preparation step. Overnight drying of 1.0 ml of raw stool homogenate in a vacuum centrifuge was performed to determine stool dry weight. Samples were then diluted to a final concentration of 2.0-mg dry weight/ml and used for sterol extraction and bile acid analysis. For SCFA analysis, an aliquot of the diluted stool homogenate was centrifuged, and 50 μl of the supernatant was subjected to derivatization to 3-nitrophenylhydrazones, prior to measurement by liquid chromatography with tandem mass spectrometry (LC–MS/MS). Sterols and stanols (coprostanol, 5α-cholestanol, sitosterol, 5α-sitostanol, 5β-sitostanol, campesterol, and 5α-campestanol) were quantified by LC–MS/MS after derivatization to N,N-dimethylglycine esters. Bile acids were quantified by LC–MS/MS using a stable isotope dilution assay with a modified method for serum. Free bile acids ursodeoxycholic acid (UDCA), chenodeoxycholic acid (CDCA), cholic acid (CA), deoxycholic acid (DCA), and lithocholic acid (LCA), as well as their glycine (G)- and taurine (T)-conjugated species were also quantified.

### Statistical analysis

The median along with the 25th and 75th percentile was used as descriptive statistics for anthropometric and biochemical/hormonal parameters; for psychometric parameters, mean and standard deviation were used. Kruskal–Wallis and post-hoc Wilcoxon rank-sum pairwise tests were applied for inter-group comparisons. Clinical data were analyzed by SPSS version 22.0 (SPSS Inc.). Two-tailed *p* ≤ 0.05 was considered statistically significant, while 0.05 < *p* ≤ 0.1 a tendency.

As for -omics data, the statistical analysis was performed by means of R Studio 1.2.1335 on R software v4.2.0 (https://www.r-project.org, last accessed on 8 June 2022) and the packages stats [51], vegan [52], and quantreg [53]. In particular, GM clusters were identified through hierarchical Ward linkage clustering based on the Spearman correlation coefficients of the proportion of OTUs, filtered by subject prevalence of at least 20%. Multiple testing using the Benjamini–Hochberg method was used to verify that each cluster showed significant correlations between samples within the group. Permutational MANOVA using the Spearman distance matrix as input, performed using the function adonis of the vegan package in R [52], was used to verify that the clusters were statistically significantly different from each other. The distribution of women by weight status within the four GM clusters was tested using Fisher’s exact test. Significant differences among the GM clusters in relative taxon abundance, alpha diversity, dietary data, and fecal lipid amounts, as well as among dietary groups (identified by application of Ward linkage clustering and Euclidean distance metrics to the first axis of a Correspondence Analysis—see the paragraph “Diet impact on the gut microbiota of normal-weight and overweight/obese women” of the “Results” section) in the healthy food diversity (HFD) index [54], were assessed using the Kruskal–Wallis test. Wilcoxon rank-sum test was adopted as a post-hoc test to check for differences between each pair of groups, adjusting *p* values for multiple testing via Benjamini–Hochberg method. Wilcoxon test with FDR correction was also used to compare alpha diversity and relative taxon abundance of the GM profiles of NW and OB women. The permutation test with pseudo-*F* ratio (function adonis in vegan) was used to assess the significance of data separation in the PCoA. The envfit function of the vegan package of R was used to perform the superimposition of FFQ data on the PCoA space, in order to identify the food items most contributing to the ordination space. For CAG assignment and analysis, see the dedicated paragraph “Co-abundance analysis” in the “Methods” section. To find associations between -omics datasets, we performed a multi-omics analysis across the entire dataset without stratifying by GM cluster. In particular, according to Chun and Keleş [55], we adopted the sparse partial least square (sPLS) regression analysis as implemented in the mixOmics package in R [56], modeling the dataset generated by metatranscriptomics (meaning transcriptionally active pathways and species) to lipidomics measures via multiple regression. sPLS is indeed a good option for sample sizes smaller than the total number of variables [55], as in our study. All lipid variables were retained in the model along with the metatranscriptomic features present in at least 50% of the samples. Hierarchical clustering on the sPLS regression model was plotted with Pearson correlation as distance and complete linkage method. Host behavioral data and other health parameters were used for correlation analysis with GM compositional data by using quantile (median) age-adjusted regression tests through the R package quantreg, as already performed by Claesson et al. [40]. *P* values were corrected for multiple comparisons using the Benjamini–Hochberg method. FDR and *p* ≤ 0.05 were considered as statistically significant.

## Results

### Study cohort description

Across two EU FP7 projects (i.e., NeuroFAST and MyNewGut), a total of 100 premenopausal women were recruited at the Unit of Endocrinology and Prevention and Care of Diabetes of the S. Orsola Polyclinic University Hospital in Bologna, Italy. Anthropometric and laboratory parameters as well as psychometric results of all recruited participants are reported in Table 1. The whole study cohort included 63 OB (with BMI from 25.6 to 39.8 kg/m^2^) and 37 NW (with BMI from 18.5 to 24.6 kg/m^2^) women. OB women showed significantly higher body weight, BMI, waist and hip circumference, and waist-to-hip ratio compared to NW (*p* < 0.001, Wilcoxon rank-sum test). Systolic and diastolic blood pressure was higher in OB compared to NW (*p* ≤ 0.001), and three OB women were under antihypertensive treatment. OB women exhibited higher total cholesterol and triglycerides levels compared to NW (*p* ≤ 0.04), while HDL-cholesterol was higher in NW group (*p* = 0.003). Glucose metabolism indices (i.e., blood glucose, insulin, glycated hemoglobin and the area under the curve (AUC) of glucose and insulin during oral glucose tolerance test) were higher in OB compared to NW women (*p* < 0.001). Five OB and one NW women had fasting glycaemia (≥ 100 mg/dl) but no one had a diagnosis of type 2 diabetes according to American Diabetes Association diagnostic criteria [54]. Finally, the OB group showed a significantly higher rate of insulin-resistant participants than NW (44.4% in OB group vs 0% in NW group, *p* < 0.001), consistent with a dependent relationship between insulin resistant/sensitive phenotype and BMI status. Correction for effect size indicated good correlation (Cramer’s correlation coefficient = 0.478, *p* < 0.0001). As for the psychometric questionnaires, some of them were discarded for incomplete data: 12 BITE (Bulimic Investigatory Test Edinburgh), 10 TFEQ (three-factors eating questionnaire), and 4 PSS (perceived stress scale) were incompletely filled in by the participants. One OB participant exhibited high scores in the two BITE subscales, indicating a high probability of fulfilling the criteria for a diagnosis of eating disorder. The mean scores obtained in the TFEQ subscales were as follows: TFEQ UE (uncontrolled eating), 16.91 ± 5.37; TFEQ CR (cognitive restraint), 14.35 ± 4.27; TFEQ EE (emotional eating), 7.59 ± 2.98. Among the 100 participants who completed YFAS, 14 (all belonging to the OB group) received a diagnosis of FA, while the mean FA symptom score of the whole cohort was 2.28 ± 1.55. None of the NW women showed high FA scores. The mean PSS score was 16.03 ± 5.91; two OB patients exhibited high PSS scores. Compared to NW, OB participants exhibited significantly higher scores in BITE severity (*p* < 0.001), TFEQ CR (*p* < 0.001), TFEQ EE (*p* < 0.001), and FA symptom score (*p* < 0.001), as well as in FA diagnosis (*p* < 0.01).

**Table 1:** Baseline characteristics of enrolled women: anthropometric, biochemical and hormonal parameters, and psychometric data. For anthropometric, biochemical, and hormonal variables, the median along with the 25^th^ percentile (p25) and 75^th^ percentile (p75) are shown for all women (All), as well as for normal-weight (NW) and overweight/obese (OB) women. For psychometric parameters, mean and standard deviation (sd) are shown. *P* values obtained by Wilcoxon rank-sum test refer to the NW vs OB comparison (significant values are in bold)

	**All**			**NW**			**OB**		
	***N***	**Median (p25-p75)**	***N***	**Median (p25-p75)**		***N***	**Median (p25-p75)**		***p***
**Anthropometric, biochemical, and hormonal parameters**									
Age (years)	100	34 (26–43)	37	33 (27–41)		63	38 (27–45)		*0.240*
Body weight (kg)	100	75.3 (62.1–84.0)	37	58.0 (53.8–64.1)		63	81.0 (76.0–90.0)		** < *0.001***
Body mass index, BMI (kg/m^2^)	100	28.1 (22.8–32.8)	37	21.6 (20.1–23.1)		63	30.9 (28.3–33.9)		** < *0.001***
Systolic blood pressure (mmHg)	99	120.0 (110.0–130.0)	37	110.0 (110.0–120.0)		62	120.0 (110.0–130.0)		***0.001***
Diastolic blood pressure (mmHg)	99	70.0 (70.0–80.0)	37	70.0 (65.0–73.0)		62	80.0 (70.0–80.0)		** < *0.001***
Waist circumference (cm)	100	95.0 (84.0–103.0)	37	80.0 (73.5–86.5)		63	101.0 (95.0–107.0)		** < *0.001***
Hip circumference (cm)	88	106.0 (97.3–115.0)	37	96.0 (91.5–100.0)		51	115.0 (107.0–121.0)		** < *0.001***
Waist-to-hip ratio	88	0.86 (0.81–0.91)	37	0.82 (0.79–0.88)		51	0.89 (0.83–0.94)		** < *0.001***
Total cholesterol (mg/dl)	100	180.0 (162.0–204.0)	37	169.0 (160.0–192.0)		63	191.0 (162.0–211.0)		***0.044***
HDL-cholesterol (mg/dl)	100	54.5 (46.3–62.8)	37	59.0 (52.5–65.0)		63	52.0 (44.0–59.0)		***0.003***
Triglycerides (mg/dl)	100	71.0 (53.3–99.8)	37	57.0 (45.5–71.0)		63	87.0 (59.0–110.0)		** < *0.001***
LDL-cholesterol (mg/dl)	97	110.0 (96.5–130.5)	37	105.0 (95.0–116.5)		60	114.0 (97.3–140.8)		*0.141*
Glycemia (mg/dl)	100	86.0 (80.0–90.0)	37	81.0 (79.0–85.5)		63	89.0 (82.0–93.0)		** < *0.001***
Glycemia-AUC (mg/dl)	98	12,930 (10,901–14,632)	37	11,130 (10,275–12,735)		61	14,085 (12,495–16,050)		** < *0.001***
Insulin (μU/ml)	100	6.8 (4.2–12.0)	37	3.7 (3.0–5.0)		63	10.1 (6.0–14.0)		** < *0.001***
Insulin-AUC (μU/ml)	97	5110 (3500–8017)	37	3412 (2472–4326)		60	7163 (4750–9966)		** < *0.001***
Glycated hemoglobin (%)	100	5.2 (4.9–5.4)	37	5.0 (4.8–5.2)		63	5.3 (5.1–5.4)		** < *0.001***
Creatinine (mg/dl)	45	0.73 (0.63–0.83)	3	0.67 (0.58–0.73)*		42	0.74 (0.64–0.84)		*0.300*
Alanine transaminase, ALT (mg/dl)	46	17.5 (12.0–24.0)	3	7.0 (6.0–24.0)*		43	18.0 (13.0–24.0)		*0.163*
Aspartate transaminase, AST (mg/dl)	46	16.0 (14.0–20.0)	3	14.0 (13.0–23.0)*		43	16.0 (14.0–20.0)		*0.643*
Uric acid (mg/dl)	43	4.3 (3.7–4.8)	3	3.4 (2.8–3.9)*		40	4.3 (3.9–4.9)		***0.020***
Erythrosedimentation rate (mm/h)	44	11.5 (6.0–19.8)	3	2.0 (2.0–4.0)*		41	13.0 (7.0–20.0)		***0.002***
C-reactive protein (mg/dl)	43	0.3 (0.1–0.5)	3	0.03 (0.03–0.07)*		40	0.3 (0.1–0.5)		***0.004***
Thyroid stimulating hormone, TSH (mUI/ml)	48	1.56 (1.30–2.11)	4	1.88 (1.48–2.94)		44	1.53 (1.11–2.11)		*0.395*
Adrenocorticotropic hormone, ACTH (pg/ml)	45	17.0 (12.0–23.0)	3	11.0 (11.0–17.0)*		42	17.5 (12.8–23.0)		*0.122*
HOMA-IR	100	1.43 (0.83–2.66)	37	0.74 (0.61–0.99)		63	2.16 (1.34–3.25)		** < *0.001***
Matsuda Index	92	5.75 (3.62–9.47)	32	10.3 (7.88–15.08)		60	4.05 (2.83–5.83)		** < *0.001***
	**N**	**Mean ± sd**	**N**	**Mean ± sd**		**N**	**Mean ± sd**		***p***
**Psychometric parameters**									
BITE severity score	88	4.48 ± 4.51	33	1.75 ± 1.27		55	6.11 ± 4.96		** < *0.001***
BITE symptom score	88	6.53 ± 5.85	33	5.39 ± 3.43		55	7.21 ± 6.85		*0.158*
TFEQ UE	90	16.94 ± 5.39	33	15.97 ± 4.93		57	17.51 ± 5.61		*0.193*
TFEQ CR	90	14.35 ± 4.29	33	12.24 ± 2.90		57	15.58 ± 4.50		** < *0.001***
TFEQ EE	90	7.56 ± 2.99	33	5.73 ± 2.22		57	8.65 ± 2.87		** < *0.001***
YFAS symptoms	100	2.28 ± 1.55	37	1.08 ± 0.54		63	2.98 ± 1.52		** < *0.001***
YFAS diagnosed addiction ^**(**^^a^^**)**^	14	14%	37	0		63	14		** < *0.001***
PSS	96	15.53 ± 6.39	35	14.2 ± 5.19		61	16.29 ± 6.91		*0.123*

^*^In groups with *N* < 4, p25 and p75 were converted to minimum and maximum (given the amount of missing data, such comparisons must be taken with caution)

^(a)^Both number and percentage of individuals with diagnosed addictive eating behavior are reported

*BITE* Bulimic Investigatory Test Edinburgh, *TFEQ* Three-factor eating questionnaire, *UE* Uncontrolled eating, *CR* Cognitive restraint, *EE* Emotional eating, *YFAS* Yale food addiction scale, *PSS* Perceived stress scale

### Gut microbiota profiling and cluster identification

**Fig. 1:**
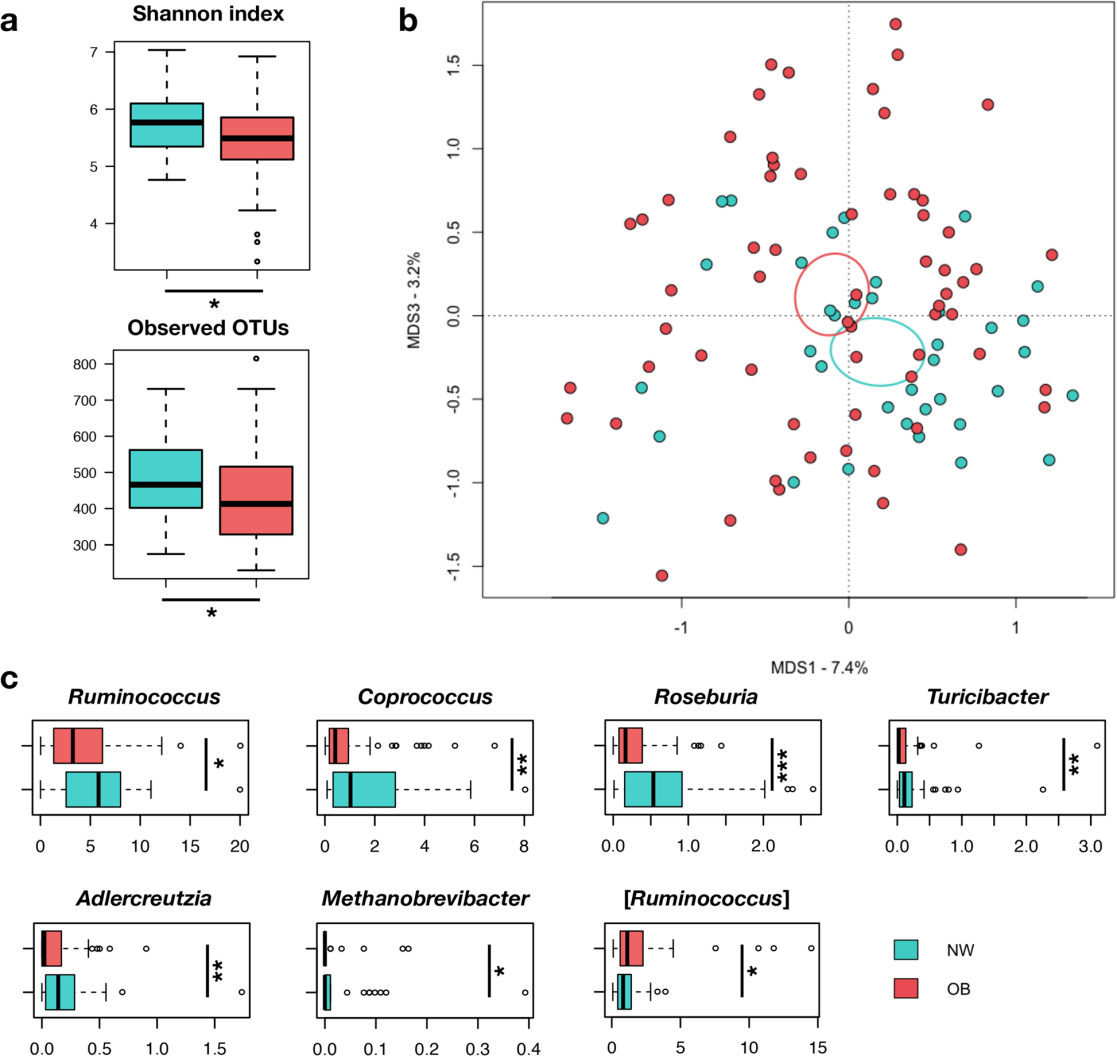
The gut microbiota structure of overweight/obese women segregates from that of normal-weight women. **a** Alpha diversity measured using the Shannon index and the number of observed OTUs for overweight/obese (OB) and normal-weight (NW) women. * *p* < 0.02, Wilcoxon rank-sum test. **b** Principal coordinates analysis (PCoA) plot based on unweighted UniFrac distances between the gut microbiota profiles of OB and NW women (*p* = 0.007, permutation test with pseudo-*F* ratio). The ellipses represent the 95% confidence interval for each study group. **c** Boxplots showing the relative abundance distribution of significantly different bacterial genera between study groups (* *p* < 0.05; ** *p* < 0.01; *** *p* < 0.001; Wilcoxon rank-sum test). Only taxa found in at least 20% of the samples were considered

16S rRNA gene sequencing yielded a total of 6.5 million sequence reads, with an average of 73,152 (± 38,578, sd) paired-end reads per sample, for 11,874 operational taxonomic units (OTUs) grouped at 97% of sequence identity. Considerable differences were identified in the GM diversity and structure of OB and NW women (Fig. 1).

**Fig. 2:**
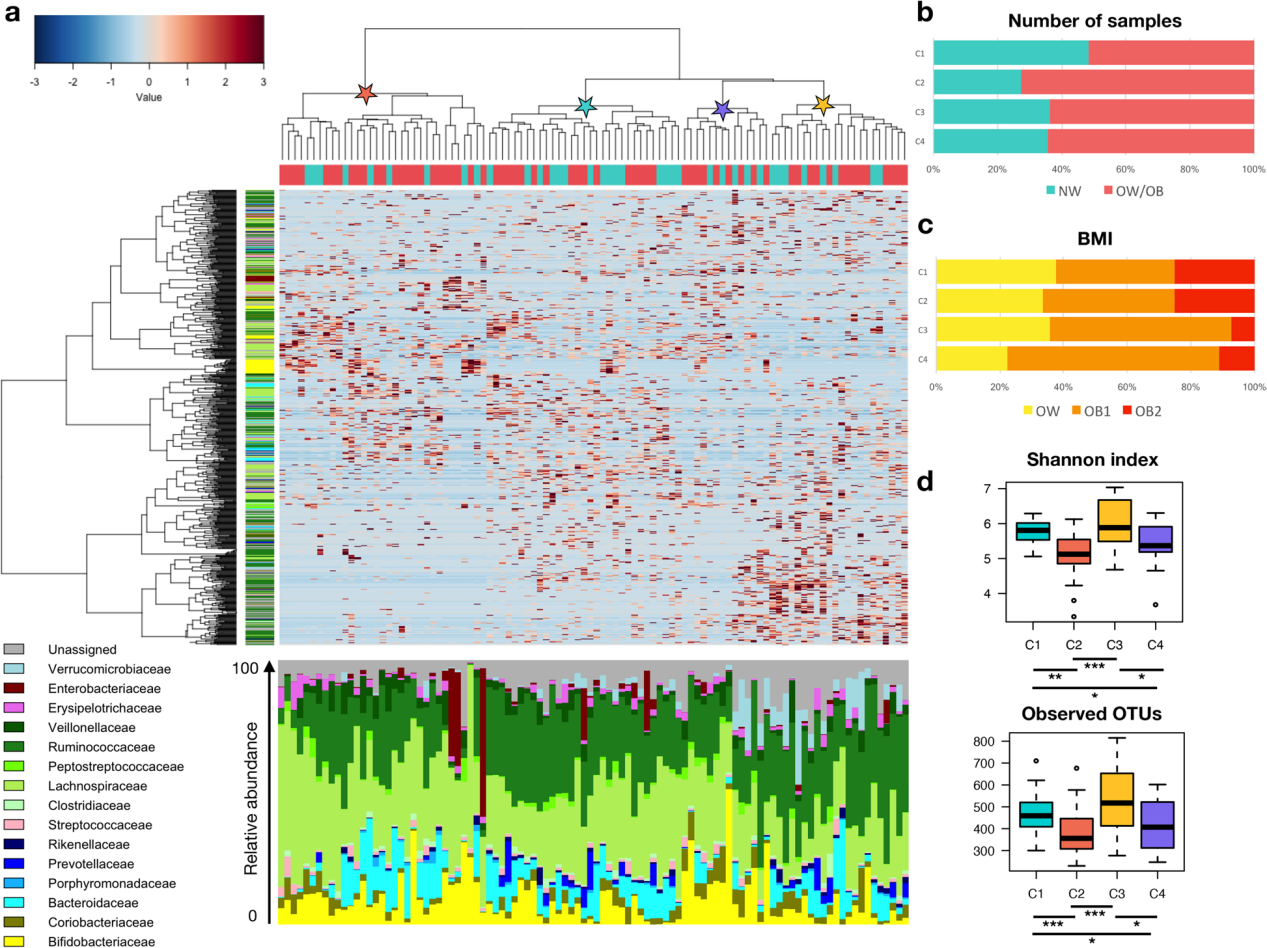
The gut microbiota structure allows stratifying the whole dataset into four distinct clusters. **a** Hierarchical Ward linkage clustering based on the Spearman correlation coefficients of the relative abundance of OTUs, filtered for presence in at least 20% of the participants. Labeled groups in the top tree (basis for the four groups of Fig. 3) are highlighted by stars colored according to the microbiota configuration (C1–C4) (see **d**). OTUs are color-coded by family assignment in the vertical tree. Bacteroidetes phylum, blue gradient; Firmicutes, green; Proteobacteria, red; and Actinobacteria, yellow. The bar plot at the bottom shows the relative abundance of the family-classified microbiota profiles. Bar plots showing the percentage of normal-weight (NW) and overweight/obese (OB) women for each cluster (**b**) and the distribution of the latter in terms of BMI status: overweight (OW), class 1 obesity (OB1), and class 2 obesity (OB2) (**c**). **d** Alpha diversity measured using the Shannon index and the number of observed OTUs for the four microbiota clusters (C1–C4). * *p* < 0.04; ** *p* < 0.001; *** *p* < 3 × 10^−4^; Wilcoxon rank-sum test

**Fig. 3:**
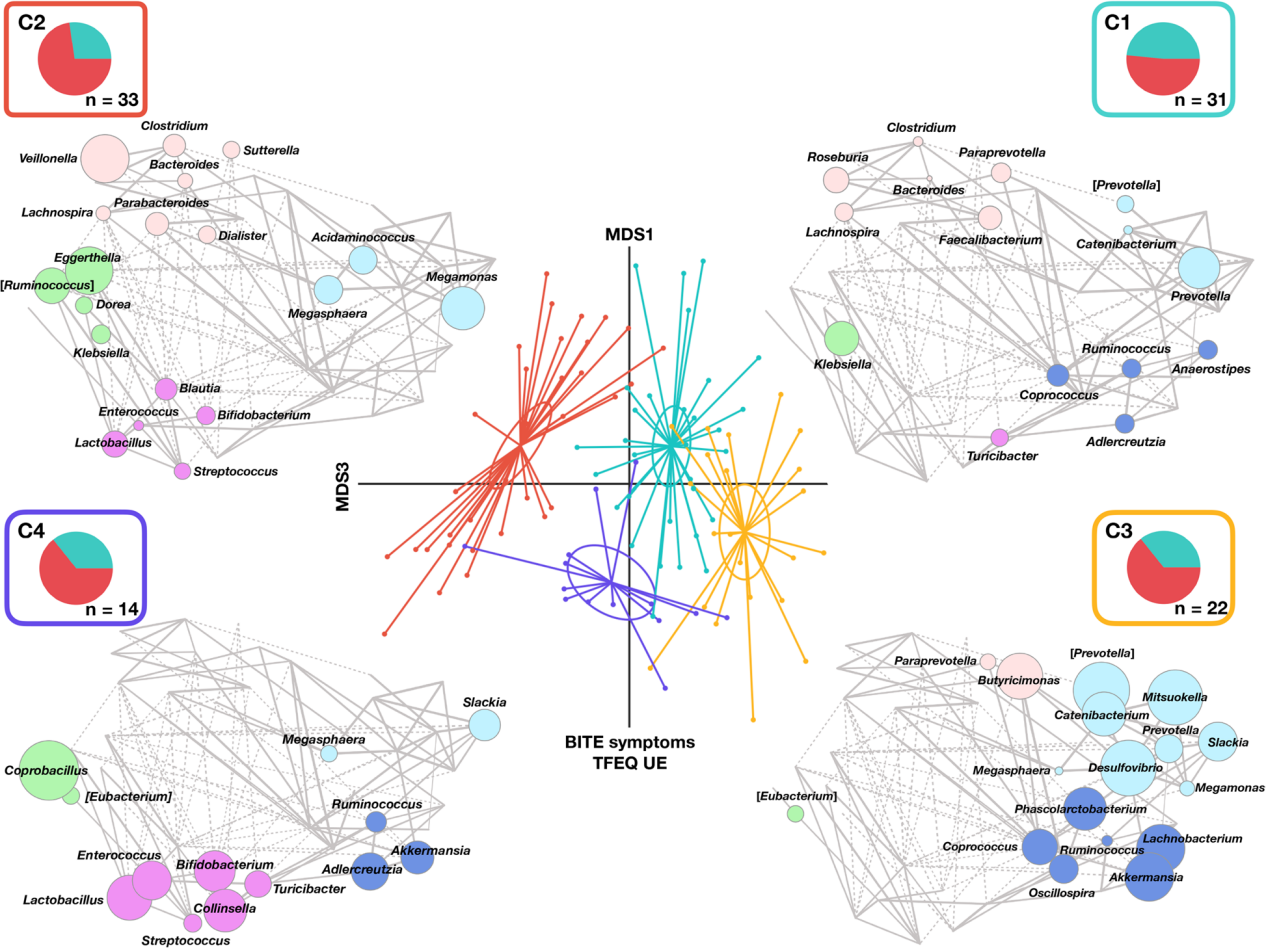
Gut microbiota structure across normal-weight and overweight/obese women is associated with eating behavior. The PCoA plot shows four significantly different clusters of participants (C1 to C4; *p* < 0.001, MANOVA), as defined by hierarchical Ward linkage clustering analysis (see also Fig. 2). Pie charts represent the distribution of normal-weight (cyan) and overweight/obese (pink) women within each cluster. For each cluster, Wiggum plots are also shown, in which disc sizes indicate genus over-abundance compared to the average relative abundance in the whole cohort (see also Additional file 1: Fig. S1). Disc colors refers to CAGs shown in Additional file 1: Fig. S1, named according to the most abundant genus in each group: *Bifidobacterium* (violet), *Ruminococcus* (blue), *Dorea* (green), *Prevotella* (light blue), and *Bacteroides* (pink). Associations with host metadata are reported (see also Table 2 and Additional file 1: Fig. S2). BITE, Bulimic Investigatory Test Edinburgh; TFEQ UE, three-factor eating questionnaire, uncontrolled eating

Hierarchical Ward linkage clustering based on the Spearman correlation coefficient of the proportion of OTUs allowed the identification of four participant clusters (named C1 to C4) (Fig. 2). Albeit in the absence of statistical significance (*p* = 0.38, Fisher’s exact test), one cluster included 48% of NW women (C1) while the remaining three clusters (C2-C4) comprised mostly OB women (72%, C2; 64%, C3 and C4). Interestingly, the four clusters also differed in biodiversity, with C1 and C3 showing the highest values, while C2 and C4 the lowest (*p* < 8 × 10^−4^, Kruskal–Wallis test). More precisely, the biodiversity in C2 was lower with respect to C1 and C3 (*p* < 3 × 10^−4^, Wilcoxon rank-sum test), while the cluster C4 showed lower levels compared to C1 (*p* = 0.05) (Fig. 2). To identify trends in the GM structure across the whole dataset, co-abundance associations of genera were first established, and correlated bacterial taxa were subsequently clustered into five co-abundance groups (CAGs) (Additional file 1: Fig. S1), named according to the dominant (i.e., the most abundant) genus in each group: *Bifidobacterium* (violet), *Ruminococcus* (blue), *Dorea* (green), *Prevotella* (light blue), and *Bacteroides* (pink). Wiggum plots were then generated to depict the GM compositional relationships for each of the four participant divisions—identified by OTU clustering—showing a peculiar abundance pattern of the five CAGs (Fig. 3). Each cluster (C1 to C4) constitutes a steady state, representing a group of individuals characterized by a GM layout significantly different from the other groups (*p* < 0.001, permutational MANOVA test on unweighted UniFrac data). However, it should be noted that the clusters did not significantly segregate in the weighted UniFrac-based PCoA (*p* > 0.05; data not shown), suggesting that the differences were not related to abundant GM components. These results were confirmed by comparisons of the relative abundances of the main genera (see Additional file 1: Table S1 for further details). The microbiota variation from the group comprising most of the NW women (i.e., C1) to the groups including predominantly OB women (C2-C4) was accompanied by distinctive CAGs dominance. Specifically, the cluster C1 was characterized by the co-presence of all 5 CAGs and a higher relative abundance of *Prevotella*, while in clusters C2-C4, the lack of at least one of the 5 CAGs identified was observed. In cluster C2, despite the absence of the *Bifidobacterium* CAG, a representation of the other four CAGs was preserved. On the other hand, cluster C3 lost the *Bifidobacterium* CAG but showed an over-representation of *Prevotella* and *Ruminococcus* CAGs. Finally, cluster C4 was characterized by a loss of *Bacteroides* while being enriched in *Bifidobacterium* CAG.

### Associations between the microbiota clusters and clinical and behavioral measures in normal-weight and overweight/obese women

Associations of demographic and clinical variables and eating behavior with the major axes of unweighted uniFrac PCoA analysis are shown in Fig. 3 and listed in Table 2 (see also Additional file 1: Fig. S2). In particular, based on a quantile (median) age-adjusted regression analysis when considering the whole cohort, a shift of the GM structure towards negative PCo2 values (as the low-diversity cluster C4) was associated with higher BITE symptom score—indicative of binge eating behavior—and TFEQ UE score—indicative of uncontrolled eating.

**Table 2:** Associations between host metadata and microbiota composition (age-adjusted dataset). Quantile (median) regression tests of association between metadata and microbiota composition as measured by unweighted UniFrac PCoA in all women. Significant associations are in bold. No significant associations were found across normal-weight and overweight/obese women

Parameter	PCo1			PCo2			PCo3		
	**RC range**	**RC sd**	***p***	**RC range**	**RC sd**	***p***	**RC range**	**RC sd**	***p***
**Unweighted UniFrac PCoA for all women**									
BITE symptoms	0.15971	0.02158	*0.8*	-1.15304	-0.28826	***0.008***	-0.44090	-0.12247	*0.2*
TFEQ UE	0.12822	0.01732	*0.9*	-1.11807	-0.27951	***0.005***	-0.50707	-0.14085	*0.2*

*RC range* Regression coefficients scaled to the full variation along each PCoA axis, thus indicating direction and magnitude of the association, *RC sd* Regression coefficients scaled to one standard deviation, *p* Quantile regression *p* value, *BITE* Bulimic Investigatory Test Edinburgh, *TFEQ UE* Three-factor eating questionnaire, uncontrolled eating

When comparing the baseline clinical conditions across the four clusters of participants, we found no difference in anthropometric data, as well as in biochemical profile and inflammatory biomarkers. All the parameters considered were similar in all groups except for uric acid, which was higher in C2 than in C4 (*p* = 0.02, Wilcoxon rank-sum test), although the values were still within the normal range (Table 3). Although statistical significance was not reached, cluster C2 participants also tended to show higher levels of triglycerides, insulin (AUC), and thyroid stimulating hormone compared to C3 or C4 (*p* ≤ 0.08). Finally, when comparing C1-C4 groups, no difference in insulin-resistant rate was found (*p* = 0.47).

**Table 3:** Comparison of anthropometric, biochemical and hormonal parameters, and psychometric data across the four gut microbiome clusters. For anthropometric, biochemical and hormonal variables, the median along with the 25^th^ percentile (p25) and 75^th^ percentile (p75) are shown. For psychometric parameters, mean and standard deviation (sd) are shown. *P* values were obtained by Kruskal–Wallis test; when significant values or trends (*p* ≤ 0.1) were obtained, post-hoc Wilcoxon rank-sum tests were performed. Significant *p* values are in bold. For YFAS diagnosed addiction, number and percentage of individuals with diagnosed addictive eating behavior are reported for each cluster (Fisher’s exact test)

		**C1**	**C2**		**C3**		**C4**		***p***
	***N***	**Median (p25-p75)**	***N***	**Median (p25-p75)**	***N***	**Median (p25-p75)**	***N***	**Median (p25-p75)**	
**Anthropometric, biochemical, and hormonal parameters**									
Age (years)	31	34 (26–42)	33	32 (26–44)	22	40 (30–45)	14	32 (27–41)	*0.959*
Body weight (kg)	31	70.5 (58.0–80.0)	33	77.8 (65.5–89.5)	22	76.5 (57.5–85.5)	14	76.5 (59.6–89.4)	*0.190*
Body mass index, BMI (kg/m^2^)	31	25.6 (21.2–30.5)	33	29.7 (24.4–33.0)	22	28.2 (21.8–31.8)	14	28.6 (22.6–32.9)	*0.160*
Systolic blood pressure (mmHg)	31	115.0 (110.0–125.0)	32	120.0 (111.3–130.0)	22	112.5 (108.8–130.0)	14	120.0 (110.0–130.0)	*0.287*
Diastolic blood pressure (mmHg)	31	70.0 (70.0–80.0)	32	77.5 (70.0–80.0)	22	70.0 (65.0–75.0)	14	80.0 (70.0–81.3)	*0.150*
Waist circumference (cm)	31	88.0 (77.0–106.0)	33	96.0 (89.0–102.5)	22	95.5 (76.8–104.0)	14	94.0 (83.0–101.3)	*0.258*
Hip circumference (cm)	28	105.0 (94.5–112.0)	27	109.0 (101.0–117.0)	20	103.0 (95.0–115.0)	13	106.0 (97.0–120.0)	*0.274*
Waist-to-hip ratio	28	0.83 (0.79–0.92)	27	0.87 (0.83–0.91)	20	0.84 (0.81–0.90)	13	0.88 (0.79–0.92)	*0.701*
Total cholesterol (mg/dl)	31	175.0 (164.0–202.0)	33	170.0 (155.5–211.5)	22	193.0 (168.3–208.8)	14	185.5 (161.8–194.0)	*0.718*
HDL-cholesterol (mg/dl)	31	58.0 (47.0–64.0)	33	53.0 (47.5–59.0)	22	56.5 (45.5–59.8)	14	56.5 (46.0–68.5)	*0.148*
Triglycerides (mg/dl)	31	69.0 (51.0–106.0)	33	85.0 (60.5–106.0)	22	70.0 (56.5–98.8)	14	63.0 (47.5–91.5)	*0.077*^*(a)*^
LDL-cholesterol (mg/dl)	30	105.5 (96.8–125.0)	33	110.0 (91.0–131.5)	20	115.0 (100.0–143.8)	14	107.0 (92.8–126.0)	*0.940*
Glycemia (mg/dl)	31	85.0 (80.0–89.0)	33	86.0 (81.0–91.0)	22	88.0 (84.8–90.8)	14	81.0 (78.5–90.3)	*0.600*
Glycemia-AUC (mg/dl)	31	12,630 (10,725–15,000)	31	13,095 (10,515–14,715)	22	13,148 (11,351–14,554)	14	12,998 (10,890–14,738)	*0.911*
Insulin (μU/ml)	31	5.9 (3.2–13.0)	33	9.0 (4.8–12.0)	22	6.0 (4.1–13.0)	14	6.0 (3.9–10.6)	*0.383*
Insulin-AUC (μU/ml)	31	4709 (2670–7095)	31	7155 (4430–9725)	21	4016 (3460–7379)	14	5264 (3735–7728)	*0.054*^*(b)*^
Glycated hemoglobin (%)	31	5.2 (4.9–5.4)	33	5.3 (5.0–5.4)	22	5.1 (4.9–5.4)	14	5.2 (4.7–5.4)	*0.371*
Creatinine (mg/dl)	10	0.72 (0.63–0.85)	20	0.75 (0.60–0.82)	9	0.77 (0.63–0.90)	6	0.71 (0.67–0.77)	*0.917*
Alanine transaminase, ALT (mg/dl)	10	19.0 (14.3–24.0)	21	18.0 (12.0–24.5)	9	17.0 (13.5–28.5)	6	14.0 (9.8–17.0)	*0.180*
Aspartate transaminase, AST (mg/dl)	10	17.0 (14.8–22.3)	21	16.0 (13.0–20.0)	9	20.0 (15.0–23.5)	6	15.0 (12.3–17.0)	*0.427*
Uric acid (mg/dl)	10	4.4 (3.7–4.8)	21	4.4 (4.0–5.1)	7	3.9 (3.6–4.3)	5	3.5 (3.4–3.9)	***0.023***^***(a)***^
C-reactive protein	9	0.4 (0.1–0.5)	21	0.3 (0.1–0.5)	8	0.2 (0.1–0.4)	5	0.8 (0.5–0.23)	*0.253*
Thyroid stimulating hormone, TSH (mUI/ml)	10	1.43 (0.37–2.11)	20	1.85 (1.51–2.38)	10	1.48 (1.22–1.78)	7	0.84 (0.80–1.59)	*0.059*^*(a)*^
Adrenocorticotropic hormone, ACTH (pg/ml)	10	21.0 (11.0–29.3)	21	17.0 (12.5–23.0)	8	16.5 (13.0–23.8)	6	14.5 (11.5–22.0)	*0.771*
HOMA-IR	31	1.24 (0.66–2.60)	33	1.91 (0.97–2.71)	22	1.37 (0.83–2.96)	14	1.17 (0.77–2.37)	*0.466*
MATSUDA Index	29	6.90 (3.55–10.10)	30	5.15 (2.90–7.93)	20	7.05 (3.83–10.35)	13	5.20 (3.90–9.45)	*0.442*
	**N**	**Mean ± sd**	**N**	**Mean ± sd**	**N**	**Mean ± sd**	**N**	**Mean ± sd**	
**Psychometric parameters**									
BITE severity score	30	4.73 ± 4.56	30	4.87 ± 5.24	19	4.31 ± 4.16	9	2.66 ± 1.73	*0.124*
BITE symptom score	30	6.23 ± 5.63	30	5.40 ± 5.66	19	7.26 ± 6.64	9	9.77 ± 4.87	*0.108*
TFEQ UE	29	17.10 ± 5.55	30	16.40 ± 5.87	19	16.95 ± 4.73	12	17.82 ± 5.21	*0.254*
TFEQ CR	29	15.31 ± 5.30	30	14.36 ± 3.79	19	13.16 ± 2.77	12	13.92 ± 4.66	*0.448*
TFEQ EE	29	7.06 ± 1.49	30	7.80 ± 2.98	19	7.42 ± 3.48	12	8.50 ± 2.35	*0.198*
YFAS symptoms	31	1.93 ± 1.41	33	2.36 ± 1.56	22	2.41 ± 1.47	14	2.64 ± 1.95	*0.968*
YFAS diagnosed addiction	2	6.4%	6	18.2%	4	18.2%	2	14.3%	*0.242*
PSS	31	14.77 ± 6.27	32	16.06 ± 6.74	21	14.81 ± 6.88	12	17.33 ± 4.94	*0.380*

^(a)^C2 vs C4

^(b)^C2 vs C3

*BITE* Bulimic Investigatory Test Edinburgh, *TFEQ* Three-factor eating questionnaire, *UE* Uncontrolled eating, *CR* Cognitive restraint, *EE* Emotional eating, *YFAS* Yale food addiction scale, *PSS* Perceived stress scale

As for the psychometric measures, the C2 cluster showed the highest BITE severity score (*p* = 0.1, Kruskal–Wallis test), while C4 the highest BITE symptom score (*p* = 0.1), consistent with the association found with the PCo2 axis (Fig. 3).

To further explore the relationship between GM clusters and eating behavior, OB women were next stratified according to the diagnosis of uncontrolled eating behavior, based on the YFAS questionnaire and taking into account the contribution of stress symptoms induced by eating behavior (see the “Methods” section for further details), in the following three groups: low addictive eating behavior (i.e., with 2 or fewer symptoms, O_LA) and high addictive eating behavior (i.e., with 3 or more symptoms) with (O_DHA) or without (O_HA) FA diagnosis (Additional file 1: Fig. S3). C1 showed the lowest proportion of O_DHA women (7%), while C2 the highest (36%). On the other hand, C1 shared similar proportions of O_HA women with cluster C2 (C1, 19% vs C2, 18%). C4 showed the highest percentage of O_HA women (36%), followed by C3 (27%). The proportion of O_LA women was the highest in C1 (26%), while similar in the other clusters (C2, 18% vs C3, 18% vs C4, 14%). None of the NW women showed uncontrolled eating behavior.

### Diet impact on the gut microbiota of normal-weight and overweight/obese women

**Fig. 4:**
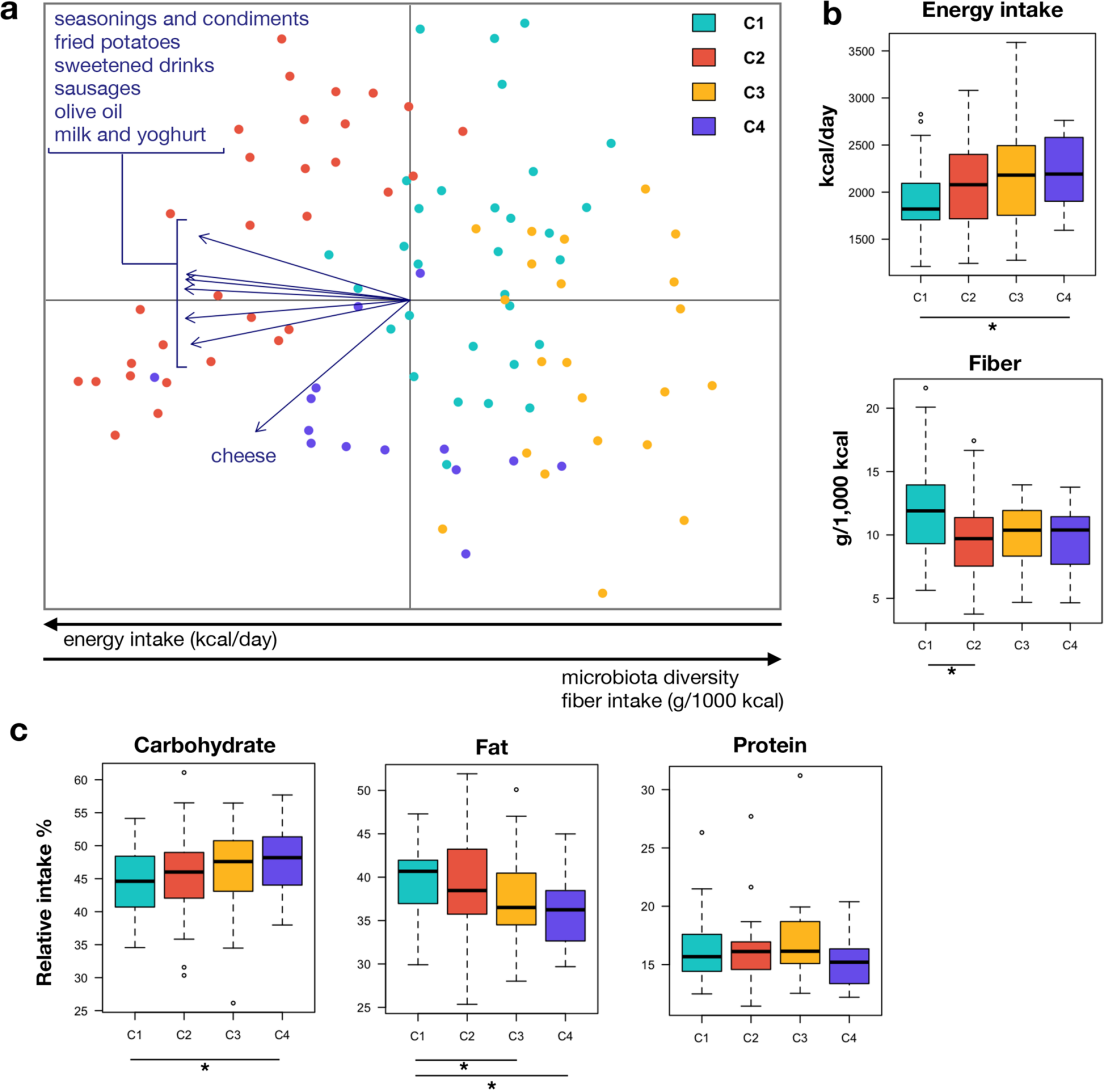
Different food consumption characterizes the different microbiota structures. **a** PCoA based on unweighted UniFrac distances of the fecal microbiota. The biplot of the average food coordinates weighted by frequency of consumption per sample was superimposed on the PCoA plot to identify the foods contributing to the ordination space (blue arrows). Only the food categories showing a significant correlation with the sample separation (*p* < 0.05, permutational correlation test) were displayed. Samples are colored by participant group (C1–C4, see Fig. 1). The black arrows at the bottom indicate the direction of the microbiota diversity, energy, and fiber intake gradient along PCo1. **b** Summary of total energy intake (in kilocalories per day) and fiber consumption (expressed as grams of fiber per 1000 kcal consumed) and **c** percentage of macronutrient intake in all women, stratified by microbiome configuration (i.e., C1 to C4). * *p* < 0.05; Wilcoxon rank-sum test

In order to identify the food types that contributed (*p* < 0.05, permutational correlation test) to the GM ordination, the food data from FFQs were superimposed on the unweighted UniFrac PCoA plot of Fig. 3 (Fig. 4a). A greater consumption of seasonings and condiments, olive oil, fried potatoes, and sausages, as well as sweetened drinks, milk, and yoghurt was associated with the GM configuration of cluster C2. On the other hand, the cluster C4 was characterized by higher consumption of cheese, while C1 and C3 by lower consumption of all the above-mentioned foods. The fiber intake (grams/1000 kcal) showed a positive correlation with the first PCoA axis and appeared to be higher in women with C1 cluster (Fig. 4b). The other three clusters showed comparable fiber intake values, with C2 being lower than C1 (*p* = 0.03, Wilcoxon rank-sum test). An opposite trend was observed for total energy intake (kcal/day), being negatively correlated to PCo1, and higher in clusters C2, C3, and C4 (Fig. 4b). Consistent with a greater propensity to uncontrolled eating (TFEQ UE) and exacerbated BITE symptom score, cluster C4 showed a higher energy intake than C1 (*p* = 0.04). The average frequency values of daily food consumption for each of the four GM groups are shown in Additional file 1: Table S2, together with additional information on each food category. When focusing on the intake of macronutrients (Fig. 4c), increased carbohydrate intake and reduced fat intake were observed in C4 compared with C1 (*p* = 0.05 and 0.04, respectively). A lower fat intake was also observed in C3 than in C1 (*p* = 0.01).

**Fig. 5:**
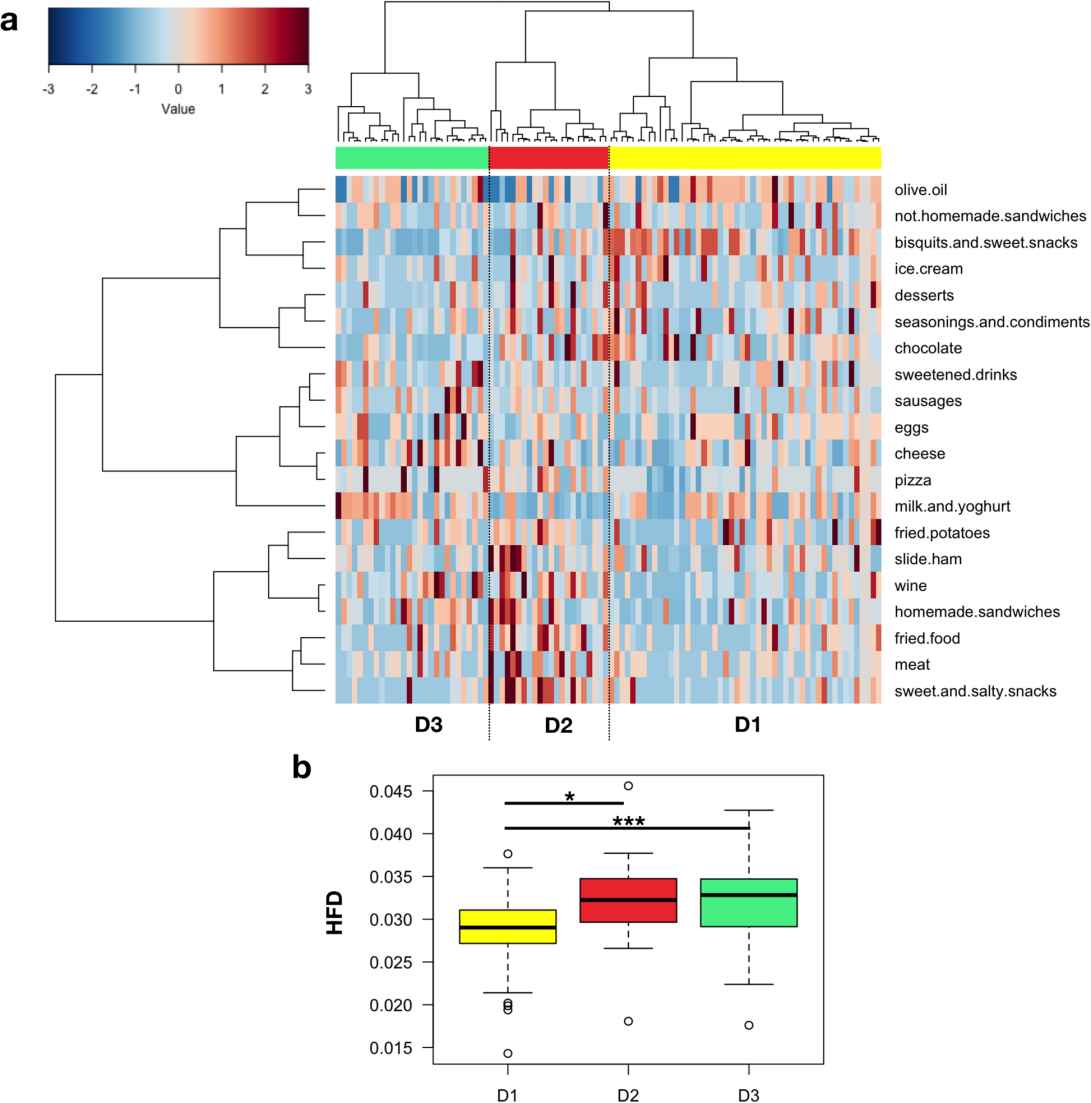
Dietary patterns discriminate women for the Healthy Food Diversity index. **a** Heat plot showing the three dietary groups (D1–D3) revealed through Ward linkage clustering using Euclidean distances applied to the first eigenvector in a Correspondence Analysis of data from Food Frequency Questionnaires. **b** Boxplots showing the distribution of the Healthy Food Diversity (HFD) index [57] across the dietary groups. * *p* = 0.02; *** *p* = 0.0001; Wilcoxon rank-sum test

FFQ data were further explored in a Correspondence Analysis, where the first axis, describing over 13.7% of the dataset variance, contained most of the discriminating food types identified in the previous correlation analysis of FFQ data on the microbiota PCoA (i.e., cheese, sweetened drinks, seasonings and condiments). Application of Ward linkage clustering and Euclidean distance metrics to this axis allowed identifying three dietary groups: D1 (“low protein/high carbohydrate”), D2 (“high protein/low carbohydrate”), and D3 (“high fat/high protein”) (Fig. 5a). In particular, D1 was characterized by a greater consumption of sweet snacks, biscuits and eggs, D2 of salty snacks, fried food, meat, sliced ham, and homemade sandwiches, while D3 of dairy products (i.e., cheese, milk and yoghurt). The healthy food diversity (HFD) index, based on the number, distribution, and health value of consumed food [57], was subsequently calculated for each dietary group. According to HFD, D2, and D3 were the most diversified diets, while D1 the least (*p* = 0.0002, Kruskal–Wallis test) (Fig. 5b).

By matching the stratifications of women in dietary and microbiota groups, redundant combinations associated with the OB phenotype were sought (Additional file 1: Table S3). In particular, the combination of the less diversified D1 diet and C2 microbiota was the most prevalent among OB women (25% of the OB dataset), especially in O_LA (14%) and O_DHA (6%) women, followed by the combinations D1-C4 (6%) in O_HA women. Interestingly, none of the OB women possessed the combination D2-C4. As for the NW sub-cohort, the three dietary groups were found to be equally distributed within the C1 microbiota configuration, with the combination D1-C1 being the most prevalent (19% of the NW dataset). Three out of five NW women with the C4 microbiota configuration (8%) were associated with the less diversified D1 diet, while the remaining two (5%) with the D2 diet.

### Species-level microbiome signatures of obesity and uncontrolled eating behavior

**Fig. 6:**
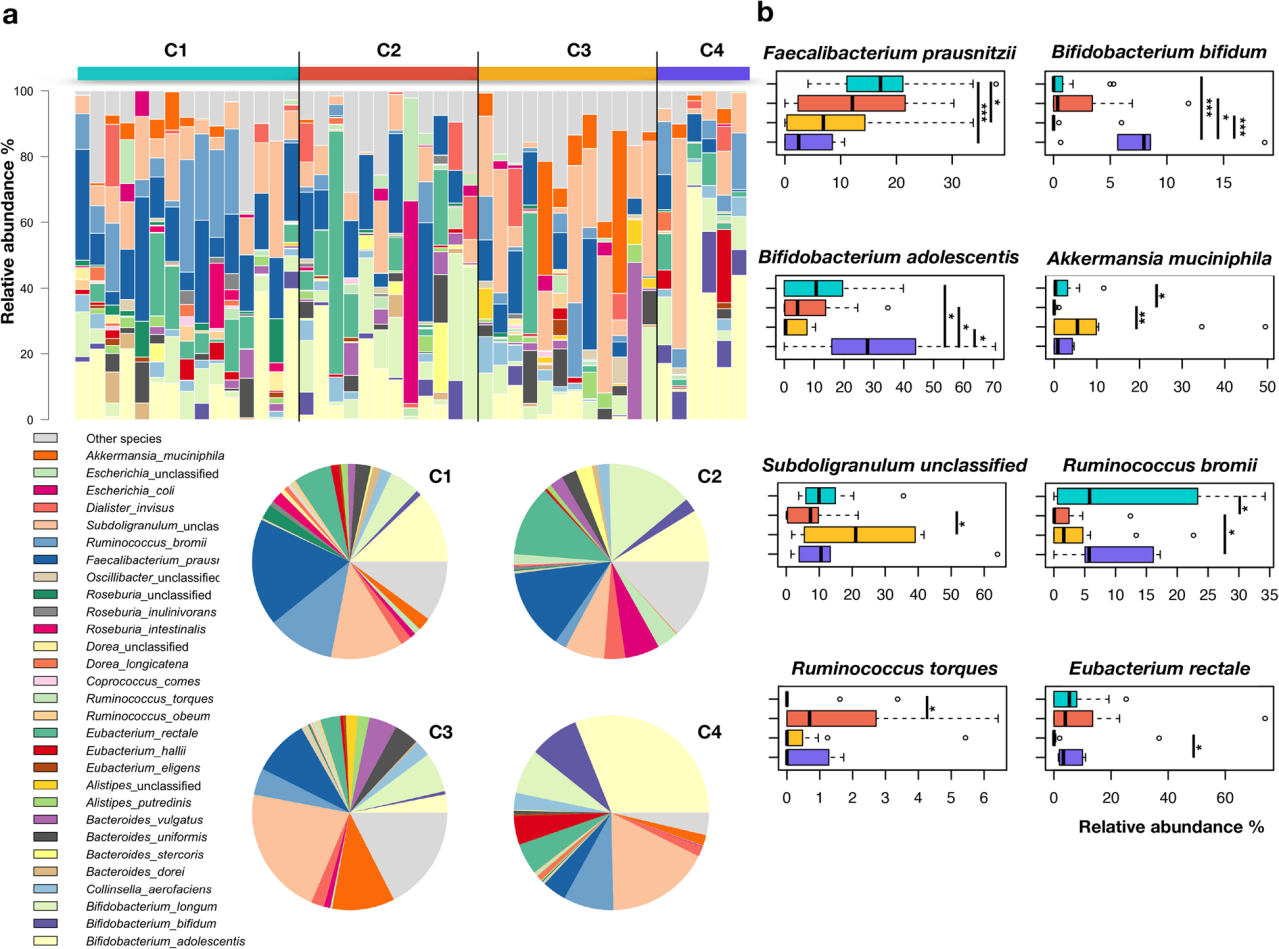
Species-level signatures of microbiome configurations. **a** Species-level relative abundance of the metagenomics profiles of the gut microbiome of enrolled women, stratified by microbiome configuration (C1–C4). Data are shown in the bar plots for each sample and in pie charts as average values. **b** Boxplots showing the distribution of relative abundances of significantly enriched or depleted bacterial species between study groups. * *p* < 0.05; ** *p* < 0.005; *** *p* < 0.0005; Wilcoxon rank-sum test

A subset of 45 DNA samples (31 from OB and 14 from NW women) was subjected to shotgun metagenomic sequencing, for a total of 15 Gb of paired-end reads. The metagenomics dataset was dominated by 8 bacterial species, which contributed 52.5–56.6% to ecosystem variability and were variously distributed among the four clusters (C1-C4): *Faecalibacterium prausnitzii*, *Bifidobacterium adolescentis*, *Bifidobacterium longum*, *Ruminococcus bromii*, *Eubacterium rectale*, *Akkermansia muciniphila*, *Bacteroides vulgatus*, and *Subdoligranulum* spp. (Fig. 6a). In particular, the cluster C1 was found to be enriched in *R. bromii* when compared to C2 (*p* = 0.03, Wilcoxon rank-sum test), as well as in *F. prausnitzii* compared to C3 and C4 (*p* < 0.03) (Fig. 6b). On the other hand, with respect to C1, the configuration C2 was enriched in *Ruminococcus torques* (*p* = 0.05), a mucolytic bacterial species known to compromise gut barrier integrity [57]. Moreover, C2 showed the lowest levels of the mucin degrader *A. muciniphila* with respect to C1 and C3 (*p* < 0.02) as well as *R. bromii* compared to C1 and C4 (*p* < 0.03). As for the other GM configurations predominantly characterizing OB women with uncontrolled eating behavior (i.e., C3 and C4), C3 showed higher values of *A. muciniphila* and *Subdoligranulum* spp. compared to C2 (*p* < 0.02), while C4 was enriched in *E. rectale* with respect to C3 (*p* = 0.008), as well as in *B. adolescentis* and *Bifidobacterium bifidum* compared to the other three clusters (*p* < 0.05), probably due to the greater consumption of cheese (as revealed by the analysis of FFQs).

### The transcriptionally active fraction of the gut microbiome and fecal lipidomic profiles in obesity and uncontrolled eating behavior

RNA sequencing was performed on the same samples subjected to metagenomics to investigate the active species-level fraction of the GM clusters and their transcriptional activity.

According to our findings (Additional file 1: Fig. S4), the most transcriptionally active fraction of the C1 configuration—mainly comprising NW women—included eight *Bacteroides* spp. (i.e., *B. faecis*, *B. finegoldii*, *B. cellulosilyticus*, *B. massiliensis*, *B. coprophilus*, *B. dorei*, *B. plebeius*, *B. vulgatus*), two *Bifidobacterium* spp. (*B. dentium* and *B. animalis*), *Coprococcus catus*, *Lachnospiraceae* bacterium (5_1_63FAA), two *Roseburia* spp. (*R. intestinalis* and *R. inulinivorans)*, and *Escherichia coli*. Regarding the configurations that included proportionately more OB women (i.e., clusters C2, C3, C4), the active microbiome fraction was found to be overall depleted of *Bacteroides* spp., while enriched in generally subdominant bacterial species (e.g., *Anaerostipes hadrus* and *Anaerostipes finegoldii*, as well as *Gordonibacter pamelaeae* in C4). More precisely, the C2 configuration—including the highest proportion of O_DHA women—showed a transcriptionally active fraction mainly composed of *A. hadrus*, four *Bacteroides* spp. (i.e., *B. thetaiotaomicron*, *B. fragilis*, *B. eggerthii*, *B. caccae*), three *Bifidobacterium* spp. (i.e., *B. pseudocatenulatum*, *B. breve*, *B. pseudolongum*), *Klebsiella pneumoniae*, *Lactobacillus ruminis*, and *Streptococcus thermophilus*. On the other hand, when compared to the other clusters (i.e., C1, C2, C4), the transcriptionally active fraction of the C3 configuration—including mostly O_HA women—was found to be the most biodiverse in terms of active species, being primarily characterized by the overabundance of three *Alistipes* spp. (i.e., *A. finegoldii*, *A. onderdonkii*, *A. shahii*), *Megamonas hypermegale*, two *Coprococcus* spp. (i.e., *C*. sp. ART55 1, *C. eutactus*), *Parabacteroides distasonis*, *Barnesiella intestinihominis*, three *Bacteroides* spp. (i.e., *B. nordii*, *B. ovatus*, *B. vulgatus*), three *Ruminococcus* spp. (i.e., *R. torques*, *R. obeum*, *R. bromii*), *R. intestinalis*, *Methanobrevibacter smithii*, two *Eubacterium* spp. (*E. eligens*, *E siraeum*), and *E. coli*. Finally, the C4 configuration—including the highest percentage of O_HA women—was found to be the least transcriptionally diversified, being characterized by few but extremely active bacterial species, including *G. pamelaeae*, *Lactobacillus casei*/*paracasei*, *B. bifidum*, *Adlercreutzia equolifaciens*, *E. rectale*, *Roseburia hominis* and *S*. *thermophilus*.

We next evaluated the core species and gene distribution within the four GM configurations, focusing on the KEGG pathways involved in carbohydrate, amino acid, lipid, and xenobiotic metabolism (Additional file 1: Fig. S4 and Fig. S5). We found that only C1 and C2 covered all the aforementioned metabolic activities with a discrete number of bacterial species. On the other hand, the C3 cluster showed a higher biodiversity but mainly related to carbohydrate and amino acid metabolism, whereas C4 was the poorest consortium, and both clusters shared almost no xenobiotic metabolism. As for the distribution of KEGG pathways, glycolysis had the highest transcript abundance and, together with nucleotide sugar and pyruvate metabolism pathways, was over-transcribed in all samples. Other metatranscriptome pathways actively transcribed across the entire dataset included the breakdown of simple sugars (e.g., fructose, mannose and galactose) and the non-oxidative pentose phosphate cycle—supporting nucleic acid synthesis—as well as essential and sulfur-containing amino acid metabolism (e.g., glycine, serine and threonine, cysteine and methionine). Regarding cluster-specific features (Additional file 1: Fig. S6), C1 metatranscriptome was particularly enriched in the abovementioned housekeeping functions, suggesting an overall efficient basal metabolism by the corresponding GM layout. On the other hand, the cluster C2 showed a peculiar high transcript abundance for propanoate metabolism. The transcriptomic landscape of C3 cluster was overall the most active and diversified, with a high abundance of transcripts involved in the metabolism of several amino acids, fatty acid biosynthesis, butanoate metabolism, and pentose/glucuronate interconversion. The C4 transcriptome showed an increased abundance of transcripts devoted to glycerolipid and glycerophospholipid metabolism, suggesting alterations in fatty acid digestion. It is also worth noting that both C3 and C4 clusters showed increased transcription of genes involved in the biosynthesis of aromatic amino acids (i.e., phenylalanine, tyrosine, tryptophan), which are known to be involved in gut-brain communication [58].

Finally, a lipidomics analysis performed on the same stool samples allowed us to identify some discriminant metabolites (Additional file 1: Fig. S7) [50]. In particular, higher SCFA (i.e., butyrate, acetate, propionate) levels were found in C1 and C2 compared to C3 and C4 (*p* ≤ 0.01, Kruskal–Wallis test). Cholesterol-to-coprostanol conversion was also reflected quite well within the former clusters. Clusters C3 and C4 were enriched in a number of converted sterols, such as coprostanol, 5β-sitostanol, and 5β-campestanol (*p* ≤ 0.04). On the other hand, the highest levels of cholesterol were associated with C2 (*p* ≤ 0.05, Wilcoxon rank-sum test). In accordance with the definition proposed by Matysik et al. [50], C2 included many non- and low-cholesterol converters, whereas many high converters and no non-converters were included in C3. As for bile acids, higher total amounts were found in C2 and C4, with the former being particularly enriched in cholic acid and chenodeoxycholic acid (*p* ≤ 0.03, Kruskal–Wallis test).

When seeking for correlations between lipidomics measures and the transcriptionally active fraction of GM (meaning both pathways and species) by means of sPLS regression (Additional file 1: Table S4 and Fig. S8), we found the strongest correlations between features of GM clusters that included predominantly OB women (especially C4). In particular, 5β-sitostanol (enriched in C3 and C4) positively correlated with the secondary bile acid biosynthesis by *F. prausnitzii* (which showed transcriptional activity for a specific gene involved in this pathway in C4—see next paragraph), as well as with *B. longum* pathways (related to galactose metabolism, glycolysis/gluconeogenesis, and cysteine and methionine metabolism, and actively transcribed across the entire dataset) and glyceroplipid metabolism by *R. bromii* (abundantly transcribed in C4 as discussed above). The latter, together with galactose metabolism by *B. longum*, also showed the strongest inverse correlations with the SCFAs acetate and propionate, consistent with their lower levels in C3 and C4.

### Transcriptional variation in microbial genes related to metabolic homeostasis

#### Food intake and energy expenditure—ClpB and bile acids

Several GM-derived metabolites and bacterial proteins have been suggested to dialogue with the brain and regulate energy intake. Among them, the chaperon protein ClpB (caseinolytic peptidase B) has been proved to mimic the anorexigenic POMC (pro-opiomelanocortin)-derived alpha-MSH (alpha-melanocyte-stimulating) hormone, well known for its ability to influence host appetite [59]. On the other hand, bile acids contribute to the regulation of host energetic homeostasis due to their role in lipid absorption, as well as by activating host receptors involved in thermogenesis [60]. We therefore specifically assessed the transcriptional levels of ClpB and enzymes involved in the microbial metabolism of bile acids across the four GM clusters (Additional file 1: Fig. S9). ClpB was actively transcribed by *A. muciniphila* only in C1 configuration (mainly including NW women), by *L. ruminis* in C2 (i.e., mainly in O_DHA women), and by *A. equolifaciens* in C1, C3 and C4 clusters. As for bile acid metabolism, we found a cluster-specific transcriptional layout for three enzymes, i.e., choloylglycine hydrolase (K01442), 7-alpha-hydroxysteroid dehydrogenase (K00076), and 3-dehydro-bile acid delta 4,6-reductase (K07007). The microbial gene coding for K01442, the primary bile acid-deconjugating enzyme, was found to be actively transcribed by several microbial components of the clusters C1 and C3, namely *R. intestinalis*, *C. catus*, *B. animalis*, *Coprococcus comes*, *Eubacterium ventriosum*, *Dorea longicatena*, and *A. shahii* for C1 and *R. obeum*, *M. smithii*, *A. finegoldii*, *P. distasonis*, *Eubacterium hallii*, *A. onderdonkii*, and *A. equolifaciens* for C3. In contrast, K01442 was exclusively transcribed by *A. hadrus* and *L. casei/paracasei* in clusters C2 and C4, respectively. As for K00076, involved in secondary bile acid biosynthesis, it was found to be exclusively transcribed by *E. coli* in C1 cluster, while no transcriptional activity was observed for the configurations that included proportionately more OB women (clusters C2-C4), regardless of eating behavior. Finally, K07007, another enzyme involved in secondary bile acid biosynthesis, was exclusively transcribed by *C. catus* and *M. smithii* in C1, by *R. obeum*, *A. shahii* and *E. rectale* in C2, and by *R. bromii*, *Coprococcus* sp. ART55_1 and *M. hypermegale* in C3. As for C4, only a weak transcriptional activity by *F. prausnitzii* and *R. hominis* was observed.

#### Neuroendocrine signaling—tryptophan metabolites, opioids, endocannabinoids, and GABA

Patterns of microbial transcriptional activity specifically involved in the metabolism of tryptophan (Trp), endocannabinoids (eCBs), and opioids, as well as in the biosynthesis of GABA, bioactive molecules able to interact with the central nervous system and influence ingestive behavior [61, 62], were subsequently investigated (Additional file 1: Fig. S9).

The clusters C1 and C4 showed comparable Trp-related transcriptional activity lower than C2 and C3, with only slight transcriptional activity responsible for the conversion of indole-3-pyruvic acid to indole-3-lactic acid (K03778), attributed to *R. intestinalis* and *R. hominis*, respectively. On the other hand, the configurations that included predominantly OB women (i.e., clusters C2, C3, C4) shared increased transcriptional activity for the conversion of pyruvate into acetyl-CoA by other genes (K00170 and K00172), although attributable to different bacterial actors. Interestingly, the C3 cluster—which mainly included O_DHA women—was characterized by the highest microbial activity devoted to directly converting Trp to indole through tryptophanase (K01667), with *A. finegoldii*, *A. muciniphila*, *A. shahii*, *B. ovatus*, and *A. onderndonkii* being the most transcriptionally active species.

With regard to the eCB system, we queried our transcriptome dataset for microbial enzymes involved in the biosynthesis of precursors of endogenous ligands of cannabinoid receptors (i.e., anandamide and 2-arachidonoylglycerol). According to our findings, the enzyme triacylglycerol lipase (K01046), involved in the formation of diacylglycerol, a precursor of 2-arachidonoylglycerol [63], was actively transcribed exclusively in C1 by *B. dentium*. C2 showed a residual activity by *B. dentium*, while no transcriptional activity was observed in C3 and C4 clusters.

As for the opioid metabolism, the C1 configuration showed the highest transcriptional levels of β-glucuronidase (K01995), the microbial enzyme involved in the metabolic pathways of morphine [64], by *B. dentium* and *B. longum*, as well as by *B. finegoldii*. On the other hand, *Bifidobacterium* and *Bacteroides* spp. were not found to be transcriptionally active in configurations that included proportionately more OB women, with only a slight activity by *E. coli* in C2, *F. prausnitzii* in C3 and *R. hominis* in C4.

Finally, the glutamate decarboxylase gene involved in GABA production (K01580) was actively transcribed in C1 by *B. faecis*, *B. cellulolyticus*, *B. uniformis*, and *B. finegoldii*. On the other hand, for the GM configurations that included predominantly OB women, the major contribution to K01580 transcription was provided by *B. dentium* and *B. fragilis* in C2, while *Bacteroides egghertii*, *B. nordii*, *B. caccae*, *B. ovatus* and *A. finegoldii* in C3. Interestingly, low to zero transcriptional activity was observed within C4.

## Discussion

To the best of our knowledge, this is the first study to use a multi-omics approach to explore associations between GM configurations, diet, and uncontrolled eating behavior in obesity, by evaluating GM composition down to the species level (including co-abundance groups—CAGs), GM transcriptional activity with particular regard to genes related to food intake, energy expenditure and neuroendocrine signaling, and fecal lipid levels. All these data were integrated with dietary intake information and various clinical and psychometric measures to provide glimpses into the gut-brain axis communication in the obese phenotype characterization.

As already observed in other studies [65, 66], we first showed that the GM of OB women has some dysbiotic peculiarities compared to NW women, i.e., a reduction in diversity and compositional alterations, including decreased proportions of typically health-associated taxa (mainly SCFA producers from the *Lachnospiraceae* and *Ruminococcaceae* families) while increased proportions of opportunistic pathogens or pathobionts (e.g., [*Ruminococcus*]).

Next, in order to gain more insights into GM layouts, in terms of steady states, we used the same approach as Rampelli et al. [7], which, by assessing the degree of similarity among GM profiles, identifies peculiar compositional clusters, each featured by distinct members and interconnections between them. Specifically, in the present study, we identified four GM clusters (C1 to C4), which differed in biodiversity, with C1 and C3 showing the highest values, while C2 and C4 the lowest, as well as in CAG abundance patterns. In particular, cluster C1 was the most diverse, with the concomitant presence of all five identified CAGs (i.e., *Bifidobacterium*, *Prevotella*, *Ruminococcus*, *Dorea*, and *Bacteroides* CAGs), while the other clusters lacked at least one CAG, namely the *Bifidobacterium* CAG for C2 and C3, and the *Bacteroides* CAG for C4. Furthermore, cluster C4 was characterized by an over-representation of *Bifidobacterium* CAG. When exploring connections with host metadata, we found that women in cluster C2 differed in higher amounts of uric acid and showed a tendency to higher levels of triglycerides, insulin, and thyroid-stimulating hormone. Regarding eating behavior, binge eating behavior and uncontrolled eating were specifically associated with cluster C4. This cluster included the highest proportion of O_HA women, i.e., with high addictive eating behavior, as defined based on the YFAS questionnaire. On the other hand, C2 included the highest percentage of O_DHA women, i.e., with FA diagnosis—taking into account the contribution of eating behavior-induced stress symptoms—while C1 the lowest.

With regard to dietary habits, we found that total energy intake was higher in clusters that included proportionately more OB women (i.e., C2 to C4) and especially in C4, consistent with the greater propensity for uncontrolled eating and exacerbated BITE symptom score. C4 was also associated with increased carbohydrate and reduced fat intake compared to C1, while the latter was characterized by the highest fiber consumption. In terms of food groups, cluster C2 was associated with greater consumption of unhealthy foods and beverages, such as fried potatoes, sausages, and sweetened drinks, while C4 with a higher intake of cheese. The relationship between GM and health-associated dietary patterns has recently been investigated in a population-level cohort, highlighting the segregation of favorable and unfavorable microbial clusters based on food source heterogeneity, quality, and dietary patterns, as well as identifying reproducible microbial indicators of obesity [67]. Our findings are also in line with the recent work by Medawar and colleagues on associations between eating behavior and dietary fiber intake, which suggested that beneficial bacteria belonging to the *Ruminococcus* genus (poorly represented in cluster C2) correlated with healthier eating behavior [68]. As recently discussed, the tendency of some GM profiles to represent more the altered metabolic phenotype of obesity (such as cluster C2 in our study) could however be related not so much to the consumption of unhealthy foods as to the absence of *Ruminococcaceae* and *Lachnospiraceae* families, which are commonly related to beneficial effects on insulin and glucose homeostasis [69, 70].

Shotgun metagenomics and metatranscriptomics were then applied with the aim of further exploring the taxonomic structure and functionality of the GM clusters identified. The former allowed us to identify some discriminating species, such as the well-known beneficial taxa *R. bromii* and *F. prausnitzii* [71, 72], which were enriched in cluster C1, and the mucus degrader *A. muciniphila*, which was most represented in C3. Identified as a next-generation probiotic candidate and extensively studied for its involvement in improving metabolic health in metabolically impaired individuals [4], the genus *Akkermansia* has recently been negatively associated with FA in OB women [73]. On the other hand, another mucolytic taxon, *R. torques*, was more present in cluster C2 (mainly including O_DHA women). Increased relative abundances of *R. torques* have been reported in patients with metabolic syndrome and inflammatory bowel disease [74]. Furthermore, this taxon has been shown to alter the microbial niche in the outer mucus layer by inhibiting the growth of *A. muciniphila* [75], as well as altering the gut barrier thus causing metabolic endotoxemia [76]. Finally, the configuration associated with the greatest proportion of O_HA women (i.e., C4) was mainly characterized by *Bifidobacterium* spp. This latter finding is apparently in contrast with the available literature, which shows that bifidobacteria can improve not only the body weight of OB women by impacting gut appetite hormones and GM composition [77], but even stress-, anxiety-, and depression-related behaviors as psychobiotics [78]. Furthermore, Kohn and colleagues have recently evaluated associations between bacterial genera and brain network connectivity, providing evidence for a relationship between bifidobacterial abundance and attention- and potentially memory-related brain network activity [79]. On the other hand, the *Bifidobacterium* overrepresentation in C4 may be associated with high cheese consumption, as discussed above.

Overall metatranscriptomic data were in line with previous ones on the compositional structure of GM clusters, i.e., that C1 and C3 were the most diverse also in terms of transcriptionally active species, while C2 and C4 were generally poor (especially C4) and tended to be transcriptionally dominated by only a few species. Furthermore, all clusters that included predominantly OB women shared an overall depletion of *Bacteroides* spp. activity. Although conflicting data have been reported on *Bacteroides* levels in obesity [80], a recent study focusing on FA in OB women highlighted that this genus was negatively associated with the connectivity of brain regions related to FA while positively with the neuroprotective metabolite, indolepropionate [73]. As for cluster peculiarities, it is worth noting that C4 showed an increased activity of *Bifidobacterium* spp. but also of other generally subdominant species, including *G. pamelae*. The latter was first isolated from the colon of a patient suffering from acute Crohn’s disease, suggesting a possible pro-inflammatory role, and found to be capable of metabolizing only a small number of carbon sources [81], which could support the association of cluster C4 with a poorly diversified diet.

Even on a functional scale, cluster C4 was the poorest, with almost no transcripts involved in xenobiotic metabolism (as in C3), and only a greater abundance of transcripts related to lipid metabolism and biosynthesis of aromatic amino acids (the latter shared with C3), whose metabolites are well known to stimulate gut-brain communication [58]. On the other hand, cluster C1 well covered all core metabolic activities and housekeeping functions (i.e., carbohydrate, amino acid, lipid and xenobiotic metabolism), consistent with its association with NW women. This was also true for cluster C2, mainly represented within O_DHA women, despite a peculiarly high abundance of transcripts for the metabolism of propanoate, a SCFA recently shown to reduce anticipatory reward responses to high-energy foods [82]. When focusing on specific microbial genes related to food intake and energy expenditure, as well as neuroendocrine signaling, we found some peculiar features that discriminated the clusters that included proportionately more OB women. In particular, genes involved in the biosynthesis of secondary bile acids were little or no transcribed in clusters C2-C4. This was supported by the lipidomics profiles [50], which showed an enrichment of primary bile acids in C2, and not unexpected given that primary bile acids are generally elevated in obesity and metabolic disorders, where they may contribute to loss of barrier function and inflammation, in addition to being closely related to the metabolic phenotype [83]. Another noteworthy finding is that numerous low- and non-cholesterol converters fell into C2, which among others lacked bacteria recently associated with cholesterol conversion (e.g., *Ruminococcus*, *Coprococcus*, and *Subdoligranulum*) [84]. Consistent with this, cluster C3 had the highest coprostanol concentrations, indicative of numerous high-converters. However, the coprostanoligenic activities of GM cluster members need to be verified and, as far as we know, no data are currently available on the possible relationship of bile acid metabolism and cholesterol conversion with uncontrolled eating behaviors. The clusters that included predominantly OB women, especially C3, were also distinguished by a greater transcriptional activity of some microbial genes linked to the production of Trp-derived indoles. Indoles are commonly reduced in OB subjects [85], but it is worth mentioning that their overproduction has recently been demonstrated to result in distinct behavioral changes in an animal model, including anxiety- and depressive-like behaviors [86], partially supporting our findings. On the other hand, cluster C2-C4 shared low mRNA levels of β-glucuronidase, an enzyme involved in the reversal of detoxification processes as well as in the morphine metabolic pathway, through the hydrolysis of the active metabolites morphine-3-glucuronide and morphine-6-glucuronide [64]. This could be related to eating disorders, as suggested by recent work showing a link between the reduction of β-glucuronidase activity and tolerance to opioids [64], which are well known to be implicated in OB-related behaviors, such as hedonic experience and binge eating [87, 88]. Finally, cluster C4 showed low to zero transcriptional activity for two genes, one involved in the biosynthesis of diacylglycerol, a precursor of 2-arachidonoylglycerol, which acts as an endogenous ligand of cannabinoid receptors, and the other involved in the production of GABA, the major inhibitory neurotransmitter in the mammalian brain. This last finding is fully consistent with the available literature, which reports reduced GABA levels in obesity and behavior alterations, such as anxiety and depression [89], and associates them with reduced inhibition of food intake in animal models [90, 91]. However, it should be noted that GABA production from glutamate decarboxylation [92] has been observed to date in a large number of so-called psychobiotics, including *Bifidobacterium* spp. [93], which were found to be overrepresented and transcriptionally active in the C4 cluster. This may suggest that such activity is not possessed by all bifidobacterial species/strains or that it is silenced in the context of certain microbial assemblages and host pathophysiological factors. As for the gene potentially related to the cannabinoid system, it is worth noting that clinical trials have shown that supplementing diacylglycerol-rich oil led to increased fat oxidation and better control of food intake by reducing the feelings of hunger, appetite and desire to eat [94]. It is therefore tempting to speculate that cluster C4, mostly represented in O_HA women, may contribute to an impaired satiety/hunger regulation system, as it is specifically poor in functions that control food intake.

In summary, through an exploratory multi-omics approach, merging data regarding GM layouts, ecological networks, transcriptional activity and lipidomic profiles with dietary intake information, and clinical and psychometric results, we showed that GM can assume a series of configurations, featured by different biodiversity, microbial actors, and functionalities, which may reflect as many aspects of the host's physiology, variously correlating with host factors, including eating habits and behaviors (Additional file 1: Table S5). In particular, OB women with high uncontrolled behavior possessed overall low-diversity GM profiles (clusters C2 and C4), dominated by a few species (*R. torques* and *Bifidobacterium* spp.), with limited transcriptional activity, especially in relation to metabolites that are known to play a crucial role in healthy gut-brain communication (e.g., secondary bile acids and GABA). Consistently, high amounts of primary bile acids as well as sterols, including cholesterol, were present in their feces. These GM clusters were also associated with increased energy intake. Nonetheless, clusters C2 and C4 showed distinctive features, such as higher uric acid, cholesterol and SCFA levels associated with the former, and higher fiber and carbohydrate intake associated with the latter, which deserve further attention for potential differential implications on human health. In contrast, NW women were characterized by a highly diverse GM layout (cluster C1), enriched in health-associated taxa, such as *R. bromii* and *F. prausnitzii*, which were overall well interconnected and transcriptionally active, covering major bacterial metabolic pathways. Cluster C1 was also characterized by high fiber consumption and high levels of SCFAs. It must be said that, although including predominantly OB women and being associated with reduced levels of SCFAs and a higher rate of converted sterols, cluster C3 shared numerous characteristics with cluster C1, suggesting a configuration that is not irreparably altered and therefore perhaps more easily redirected. Despite the considerable amount of information gathered and the interesting results emerged, some limitations should be mentioned: the participants had a screening visit for psychiatric disorders based on the MINI interview and completed a battery of psychometric questionnaires, but nevertheless, the presence of symptoms of psychological distress could be completely ruled out; the small sample size (especially of OB women in the O-HA group); the potential inaccuracy related to self-reported FFQs; the lack of significance of the distribution of NW/OB women across GM clusters; the lack of availability of clinical data for all women enrolled, which likely contributed to the failure to identify cluster-specific clinical features; the exploratory association analysis approach and the high probability of false positives for multiple inference tests; the lack of use of a more exhaustive metabolomics approach to assess other molecules possibly contributed by GM in multiple biological samples; and the lack of mechanistic validation.

## Conclusions

Our exploratory study allowed us to generate compelling hypotheses on the system biology related to OB and uncontrolled eating behavior, by showing that peculiar compositional and functional structures of GM are strongly associated with eating habits and behaviors. In particular, the potential GM signatures found in OB women with high uncontrolled behavior suggest the involvement of a low-diversity ecosystem, dominated by few microorganisms, and characterized by a poor transcriptional and lipidomic landscape, which could negatively affect gut-brain communication. Our findings should be verified in larger patient cohorts and integrated with animal experiments to elucidate the underlying mechanisms. Once confirmed, these findings could pave the way for the implementation of precision microbiome-tailored intervention strategies, mainly aimed at recovering diversity, understood as microbial components, ecological interactions, and functions, for a diet-GM-gut-brain axis that promotes healthy behaviors and prevents OB-related complications. 

## Supplementary Information

**Additional file 1: Fig. S1.** Assignment of bacterial co-abundance groups (CAGs). **Fig. S2.** Associations of the microbiota profiles with host metadata. **Fig. S3.** Distribution of eating behavior-based study groups across the four microbiome clusters (C1-C4).** Fig. S4.** The transcriptionally active fraction of the gut microbiome differs according to obesity and uncontrolled eating behavior. **a** Bubble charts showing the transcriptionally active microbial species within the four microbiome configurations (clusters C1–C4). Circle sizes and colors indicate the transcriptional over-abundance of a given species compared to its average normalized abundance in the whole cohort using log_2_ transformation. **b** Spider plots showing the number of active species for each metabolism (i.e., carbohydrate, lipid, amino acid and xenobiotic metabolism). Species were counted as “active” when present with log_2_ normalized abundance > 1 in at least 1/3 of the pathways comprised in the specific metabolic category. ** Fig. S5.** The metabolic activities of the gut microbiome are broadly taxonomically distributed. **Fig. S6.** Metabolic activities of the four microbiota clusters according to metatranscriptomic analysis. **Fig. S7.** Fecal lipidomic profiles of the four microbiota clusters (C1-C4). **Fig. S8.** Associations between the transcriptionally active fraction of the gut microbiome and lipidomics measures. Hierarchical clustering obtained with complete linkage method and Pearson correlation as distance, performed on the sparse partial least square (sPLS) regression of transcriptionally active pathways and species of the gut microbiome and host lipids. CA, cholic acid; CDCA, chenodeoxycholic acid; DCA, deoxycholic acid; GCA, glycocholic acid; GDCA, glycodeoxycholic acid; HDCA, hyodeoxycholic acid; LCA, lithocholic acid; UDCA, ursodeoxycholic acid. **Fig. S9.** Transcriptional activities of microbiome configurations specifically related to food intake, energy expenditure, and neuroendocrine signaling. Average RNA:DNA ratio for each cluster (C1–C4) with respect to the whole cohort, of a selection of key genes involved in ClpB (orange), bile acid (brown), tryptophan (green), opioid (cyan), eCB (violet), and GABA (navy) production, divided by the assigned microbial species. Phylogenetic tree on the top of the heatmap was built using hierarchical Ward linkage clustering based on the Spearman correlation coefficients of the average DNA-normalized transcript abundance profiles for the species with detectable transcriptomes in the whole cohort**Table S1.** Genus-level distribution and differences in the gut microbiota profiles among the four clusters (C1-C4). **Table S2.** Food description and average frequency of daily consumption for each microbiota cluster (C1 to C4). **Table S3.** Summary of dietary groups and microbiota clusters for each of the 100 enrolled women. **Table S4.** Loading scores obtained from sPLS correlation analysis between metatranscriptomics and lipidomics datasets. **Table S5.** Summary of the main distinctive features of the four microbiota clusters in terms of clinical, dietary and lipidomic profiles.

## Data Availability

Raw sequencing reads are available in the National Center for Biotechnology Information Sequence Read Archive (BioProject ID: PRJNA832282, PRJNA832560, PRJNA832581).

## Abbreviations

BITE: Bulimic Investigatory Test Edinburgh
BMI: Body mass index
CAG: Co-abundance group
ClpB: Caseinolytic peptidase B
CR: Cognitive restraint
eCB: Endocannabinoid
EE: Emotional eating
FA: Food addiction
FDR: False discovery rate
FFQ: Food Frequency Questionnaire
GM: Gut microbiome
MINI: Mini-International Neuropsychiatric Interview
MSH: Melanocyte-stimulating hormone
NW: Normal-weight
OB: Overweight/obese
O_DHA: High addictive eating behavior, including participants with three or more symptoms and FA diagnosis
O_HA: High addictive eating behavior, including participants with three or more symptoms but no FA diagnosis
O_LA: Low addictive eating behavior, including participants with two or less symptoms
PCoA: Principal coordinates analysis
POMC: Pro-opiomelanocortin
PSS: Perceived Stress Scale
SCFA: Short-chain fatty acid
TFEQ: Three-factors eating questionnaire
Trp: Tryptophan
UE: Uncontrolled eating
YFAS: Yale Food Addiction Scale
