# Supplementary material for: Multi-omics gut microbiome signatures in obese women: role of diet and uncontrolled eating behavior
Source: BMC Med. 2022 Dec 27;20:500. doi: 10.1186/s12916-022-02689-3 (PMC9795652; doi:10.1186/s12916-022-02689-3)
Supplement: Supplementary file 1 — Additional file 1: Fig. S1. Assignment of bacterial co-abundance groups (CAGs). Fig. S2. Associations of the microbiota profiles with host metadata. Fig. S3. Distribution of eating behavior-based study groups across the four microbiome clusters (C1-C4). Fig. S4. The transcriptionally active fraction of the gut microbiome differs according to obesity and uncontrolled eating behavior. a Bubble charts showing the transcriptionally active microbial species within the four microbiome configurations (clusters C1–C4). Circle sizes and colors indicate the transcriptional over-abundance of a given species compared to its average normalized abundance in the whole cohort using log2 transformation. b Spider plots showing the number of active species for each metabolism (i.e., carbohydrate, lipid, amino acid and xenobiotic metabolism). Species were counted as “active” when present with log2 normalized abundance > 1 in at least 1/3 of the pathways comprised in the specific metabolic category. Fig. S5. The metabolic activities of the gut microbiome are broadly taxonomically distributed. Fig. S6. Metabolic activities of the four microbiota clusters according to metatranscriptomic analysis. Fig. S7. Fecal lipidomic profiles of the four microbiota clusters (C1-C4). Fig. S8. Associations between the transcriptionally active fraction of the gut microbiome and lipidomics measures. Hierarchical clustering obtained with complete linkage method and Pearson correlation as distance, performed on the sparse partial least square (sPLS) regression of transcriptionally active pathways and species of the gut microbiome and host lipids. CA, cholic acid; CDCA, chenodeoxycholic acid; DCA, deoxycholic acid; GCA, glycocholic acid; GDCA, glycodeoxycholic acid; HDCA, hyodeoxycholic acid; LCA, lithocholic acid; UDCA, ursodeoxycholic acid. Fig. S9. Transcriptional activities of microbiome configurations specifically related to food intake, energy expenditure, and neuroendocrine signaling. Average RNA:DNA [file 12916_2022_2689_MOESM1_ESM.pdf]

Multi-omics gut microbiome signatures in obese women: role of diet and uncontrolled eating behavior

Monica Barone,[a,b†] Silvia Garelli,[c†] Simone Rampelli,[b] Alessandro Agostini,[d] Silke Matysik,[e] Federica D'Amico,[a,b] Sabrina Krautbauer,[e] Roberta Mazza,[f] Nicola Salituro,[c] Flaminia Fanelli,[c] Patricia Iozzo,[g] Yolanda Sanz,[h] Marco Candela,[b] Patrizia Brigidi,[a] Uberto Pagotto,[c] Silvia Turroni[b,*]

[a]*Microbiomics Unit, Department of Medical and Surgical Sciences, University of Bologna, Bologna 40138, Italy*

[b]*Unit of Microbiome Science and Biotechnology, Department of Pharmacy and Biotechnology, University of Bologna, Bologna 40126, Italy*

[c]*Unit of Endocrinology and Prevention and Care of Diabetes, Center for Applied Biomedical Research, S. Orsola Polyclinic, Istituto di Ricovero e Cure a Carattere Scientifico (IRCCS), Department of Medical and Surgical Sciences, University of Bologna, Bologna 40138, Italy*

[d]*Department of Experimental, Diagnostic, and Specialty Medicine, S. Orsola Polyclinic, Istituto di Ricovero e Cure a Carattere Scientifico (IRCCS), University of Bologna, Bologna 40138, Italy*

[e]*Institute of Clinical Chemistry and Laboratory Medicine, University Hospital Regensburg, Regensburg 93053, Germany*

[f]*Present address: Research Development - Life Sciences and Bioeconomy Unit, Research Services Division (ARIC), University of Bologna, Bologna 40126, Italy*

[g]*Institute of Clinical Physiology, National Research Council, Pisa 56124, Italy*

[h]*Microbial Ecology, Nutrition & Health Research Unit, Institute of Agrochemistry and Food Technology, Spanish National Research Council (IATA-CSIC), Valencia 46980, Spain*

[†] These authors contributed equally to this work.

[*] Address correspondence to Dr. Silvia Turroni, silvia.turroni@unibo.it

Supplementary Material

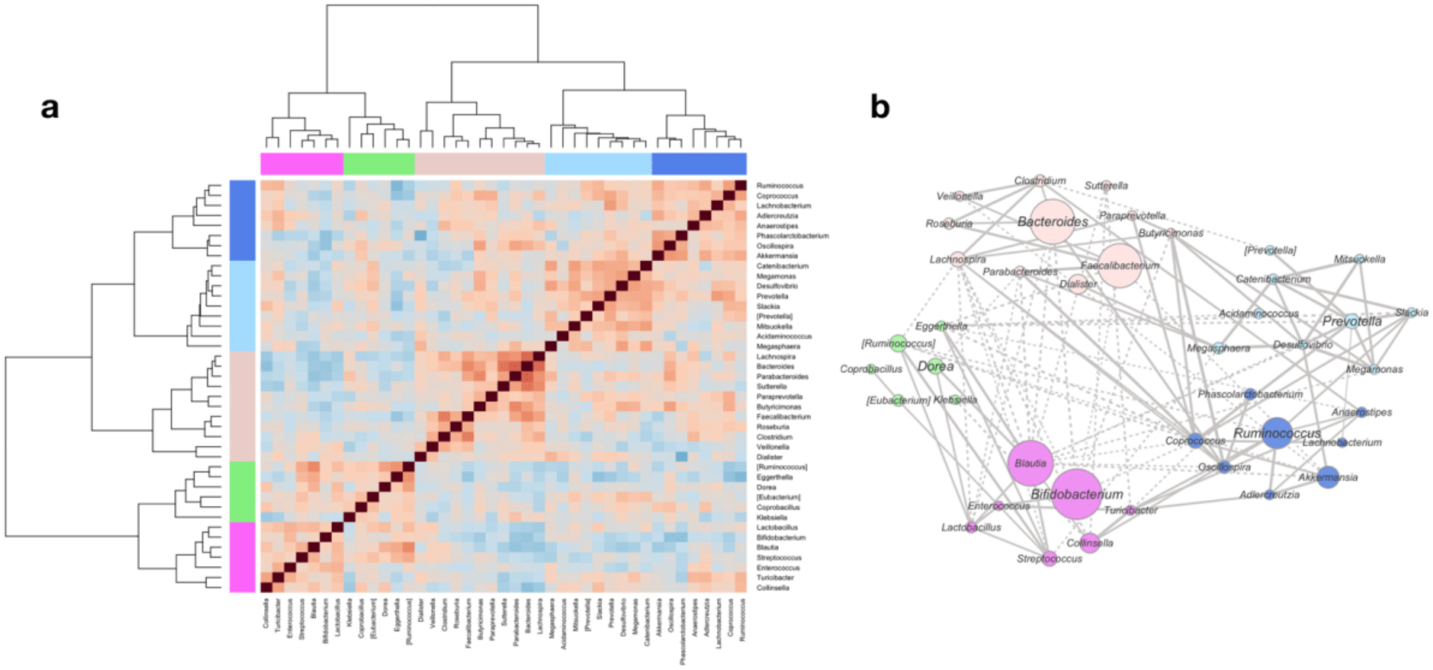

Figure S1. Assignment of bacterial co-abundance groups (CAGs). CAGs were assigned according to the heat plot **(a)** showing Kendall correlations between genera clustered by the Spearman correlation coefficient and Ward linkage hierarchical clustering. **(b)** Wiggum plot correlation between the five CAGs identified. Circle size is proportional to the genus relative abundance and the connections between nodes represent significant correlations between genera (Kendall, FDR < 0.05). Positive correlations are represented by continuous lines, while negative correlations are represented by dashed lines. Thickness of the lines is proportional to the significance of the correlation.

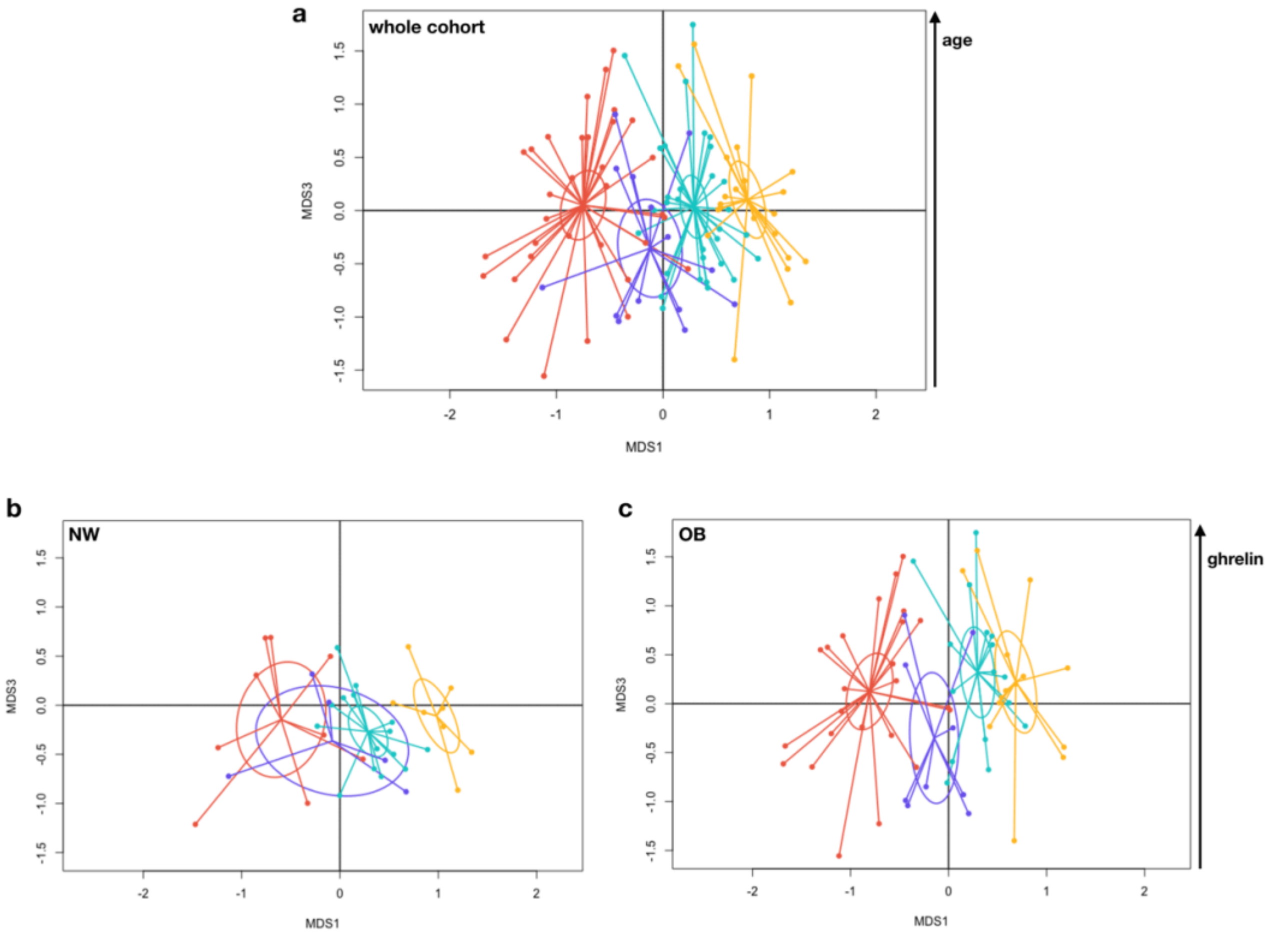

Figure S2. Associations of the microbiota profiles with host metadata. Unweighted UniFrac PCoA of the fecal microbiota in the whole cohort (**a**), as well as in normal-weight women (NW, **b**) and overweight/obese women (OB, **c**), stratified according to the four microbiota clusters (C1-C4, see Fig. 3). The host variables significantly associated with the axes are highlighted. MDS, multidimensional scaling.

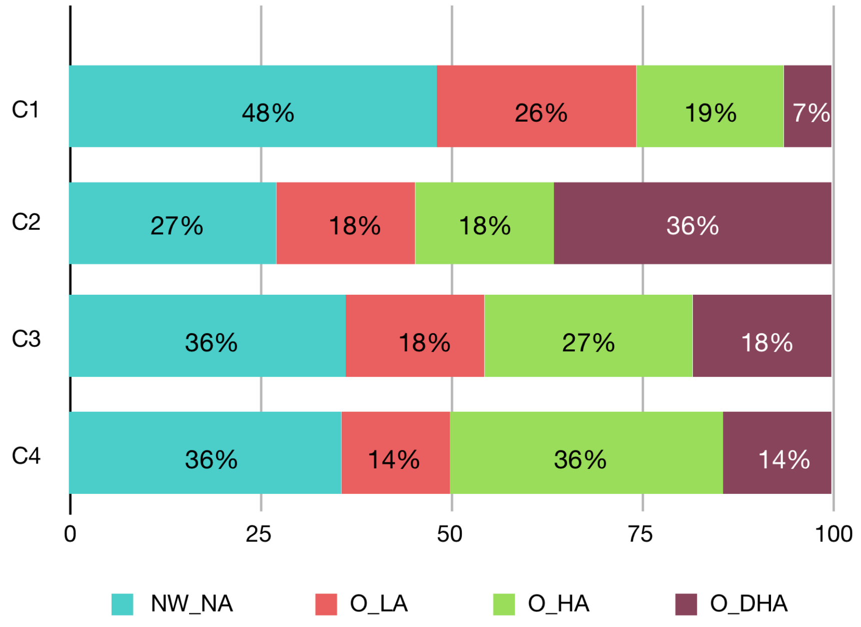

Figure S3. Distribution of eating behavior-based study groups across the four microbiome clusters (C1-C4). Women were stratified based on addictive eating behavior into 4 groups: NW_NA (normal-weight, non-addicted), O_LA (overweight/obese, low-addicted), O_HA (overweight/obese, high-addicted), and O_DHA (overweight/obese, high-addicted with food addiction diagnosis). See Methods for further details on stratification.

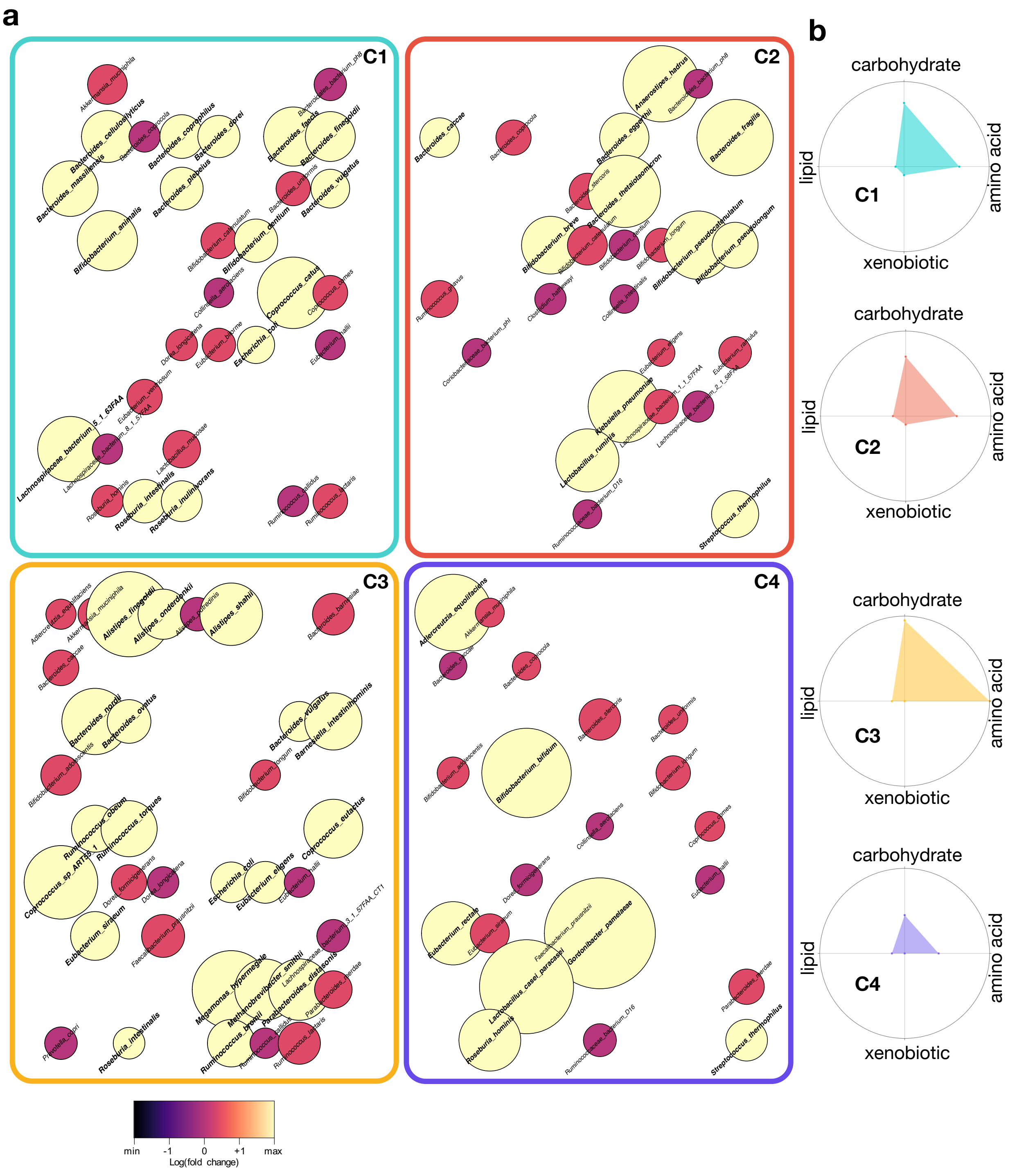

Figure S4. The transcriptionally active fraction of the gut microbiome differs according to obesity and uncontrolled eating behavior. (a) Bubble charts showing the transcriptionally active microbial species within the four microbiome configurations (clusters C1-C4). Circle sizes and colors indicate the transcriptional over-abundance of a given species compared to its average normalized abundance in the whole cohort using log2 transformation. (b) Spider plots showing the number of active species for each metabolism (i.e., carbohydrate, lipid, amino acid and xenobiotic metabolism). Species were counted as "active" when present with log2 normalized abundance > 1 in at least 1/3 of the pathways comprised in the specific metabolic category.

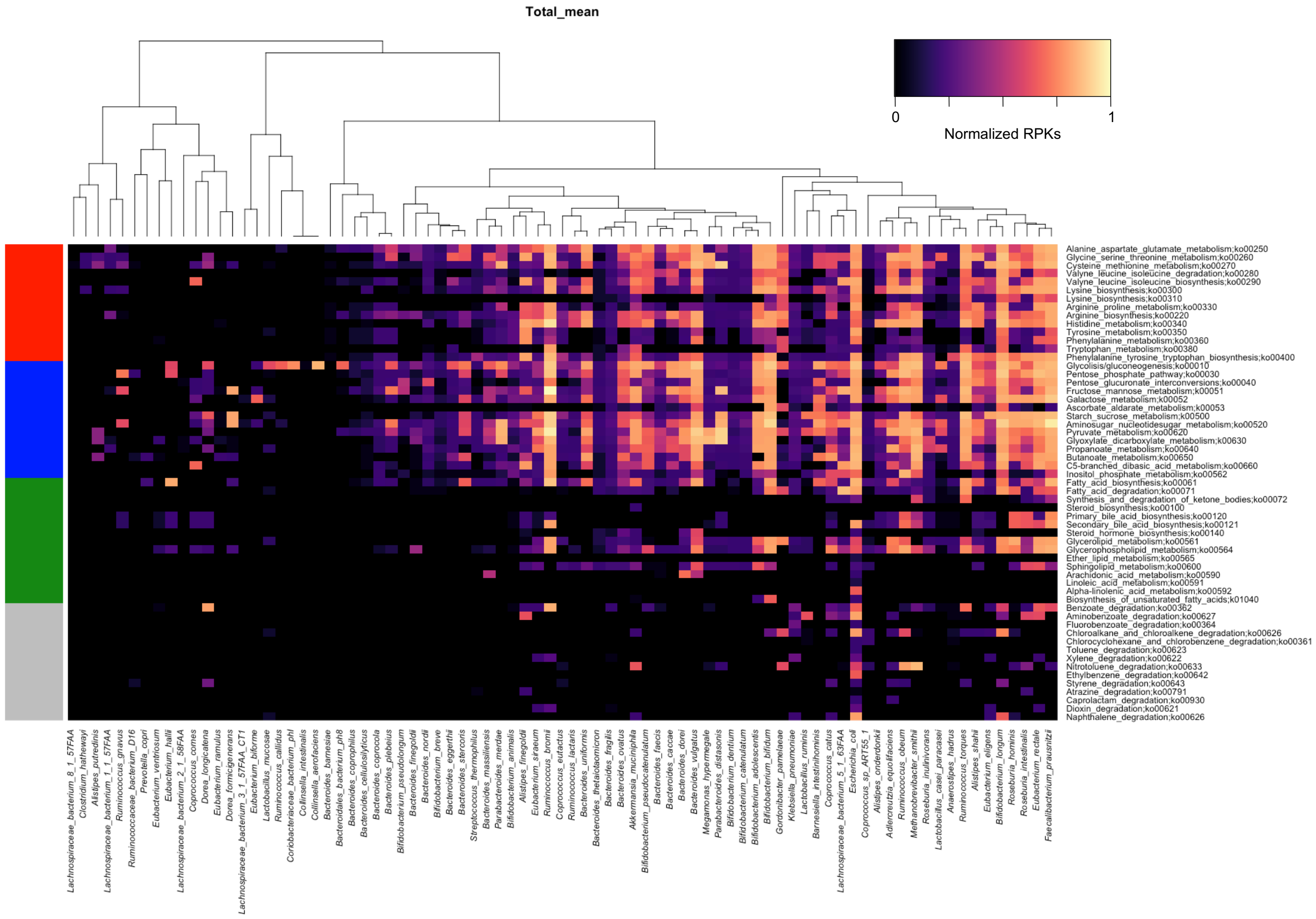

Figure S5. The metabolic activities of the gut microbiome are broadly taxonomically distributed. Transcription ratios of 57 KEGG pathways related to amino acid (red), carbohydrate (blue), lipid (green), and xenobiotic (grey) metabolisms that were most prevalently transcribed among the species detected by metagenomics. A given pathway-species combination in the heatmap represents the transcript abundance averaged over all samples that measured an RNA/DNA ratio for that species. All the pathway-species combinations in at least 1 sample were considered. Columns in the heatmap were ordered based on Ward linkage clustering on a Spearman distance matrix of DNA-normalized transcript abundance profiles for each species. Normalized RPKs correspond to DNA-normalized (RNA:DNA ratio) transcript reads per kilobases.

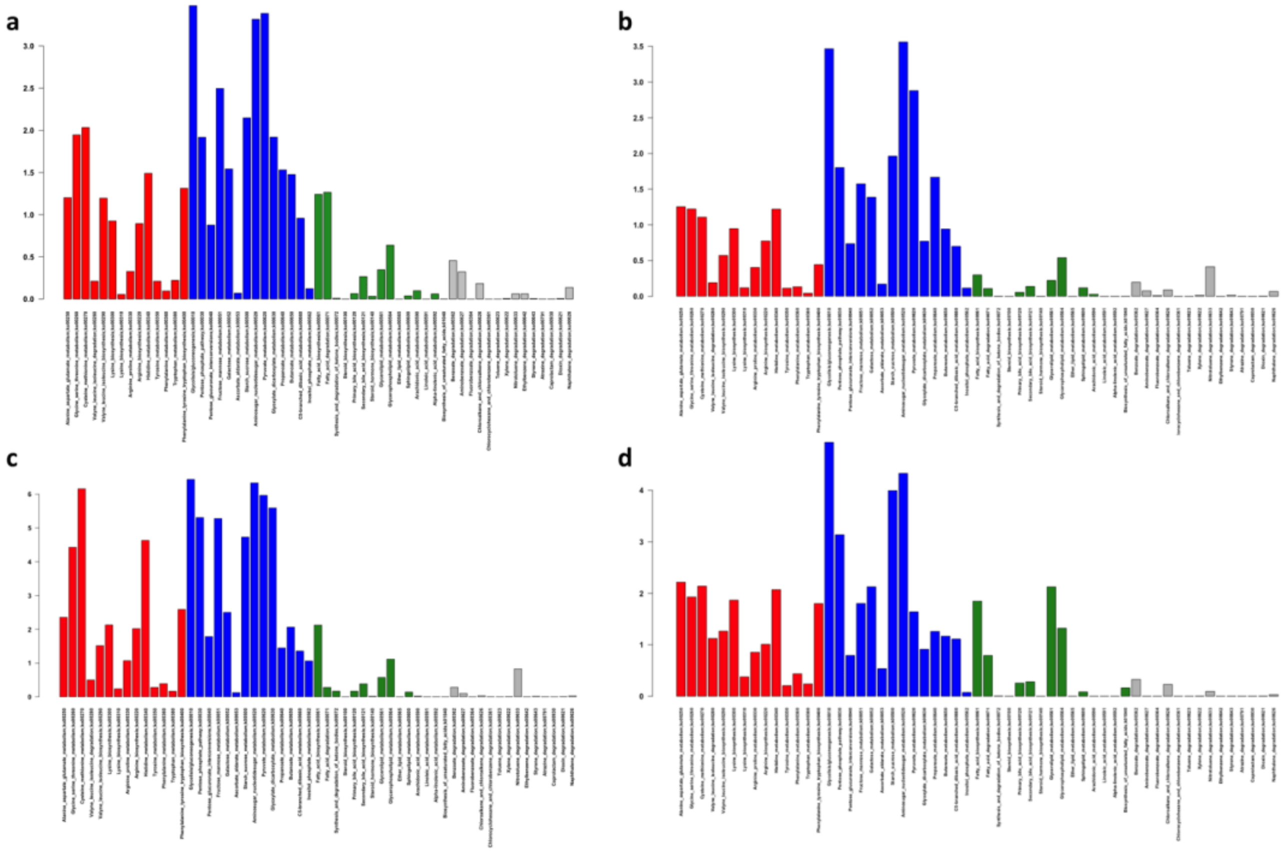

Figure S6. Metabolic activities of the four microbiota clusters according to metatranscriptomic analysis. Expression levels of the 57 identified KEGG pathways related to amino acid (red), carbohydrate (blue), lipid (green), and xenobiotic (grey) metabolisms that were most prevalently transcribed among the species detected by metagenomics, for the microbiome clusters C1 (**a**), C2 (**b**), C3 (**c**) and C4 (**d**).

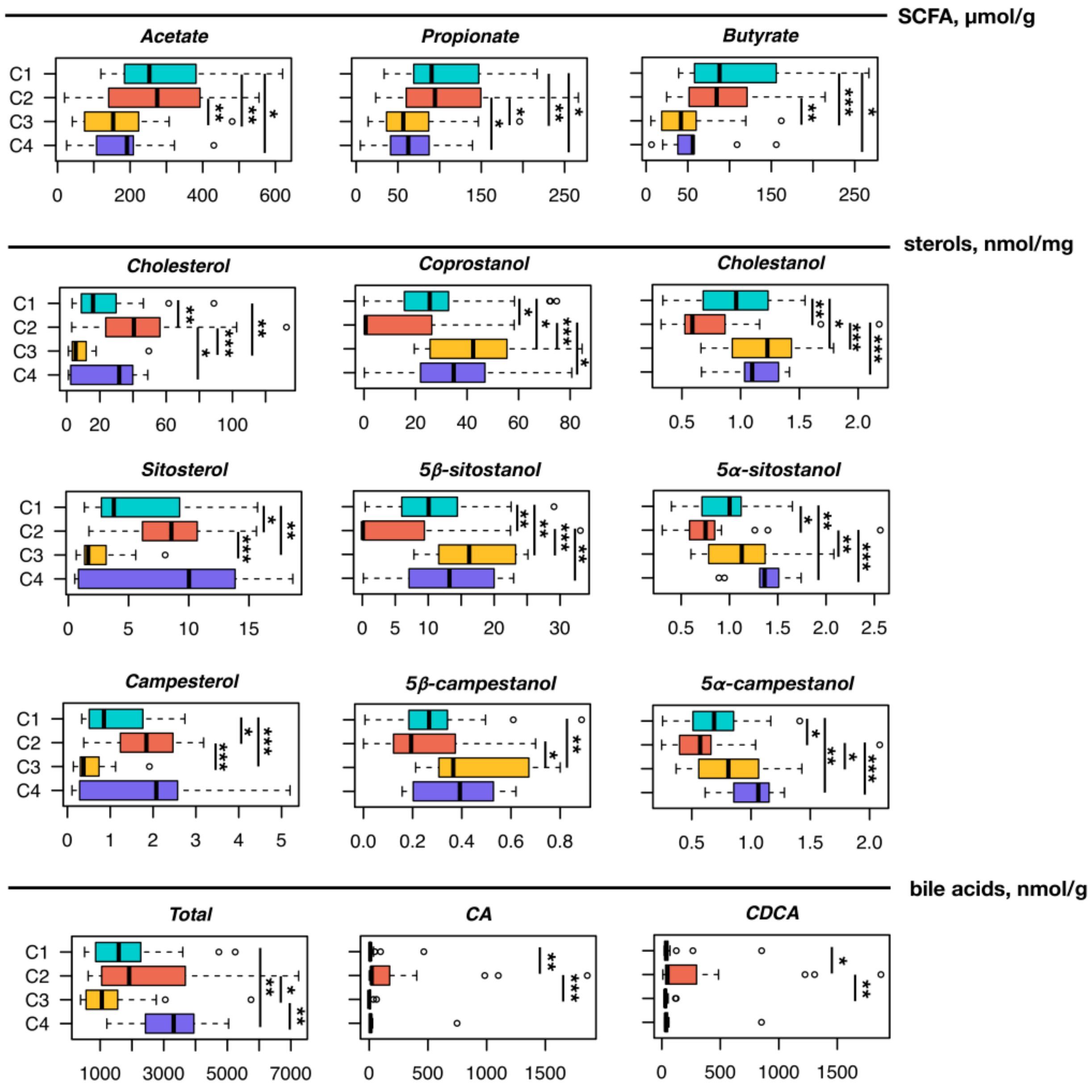

Figure S7. Fecal lipidomic profiles of the four microbiota clusters (C1-C4). Boxplots showing the amount distribution of short-chain fatty acids (SCFAs, upper panel), sterols (middle panel) and bile acids (lower panel) significantly different between the four microbiota clusters. CA, cholic acid; CDCA, chenodeoxycholic acid; GDCA, glycocholic acid; Glyco, glycoconjugates. See [50] for further details on fecal lipidomics analysis. *, $p \leq 0.05$; **, $p \leq 0.01$; ***, $p \leq 0.001$; Wilcoxon test.

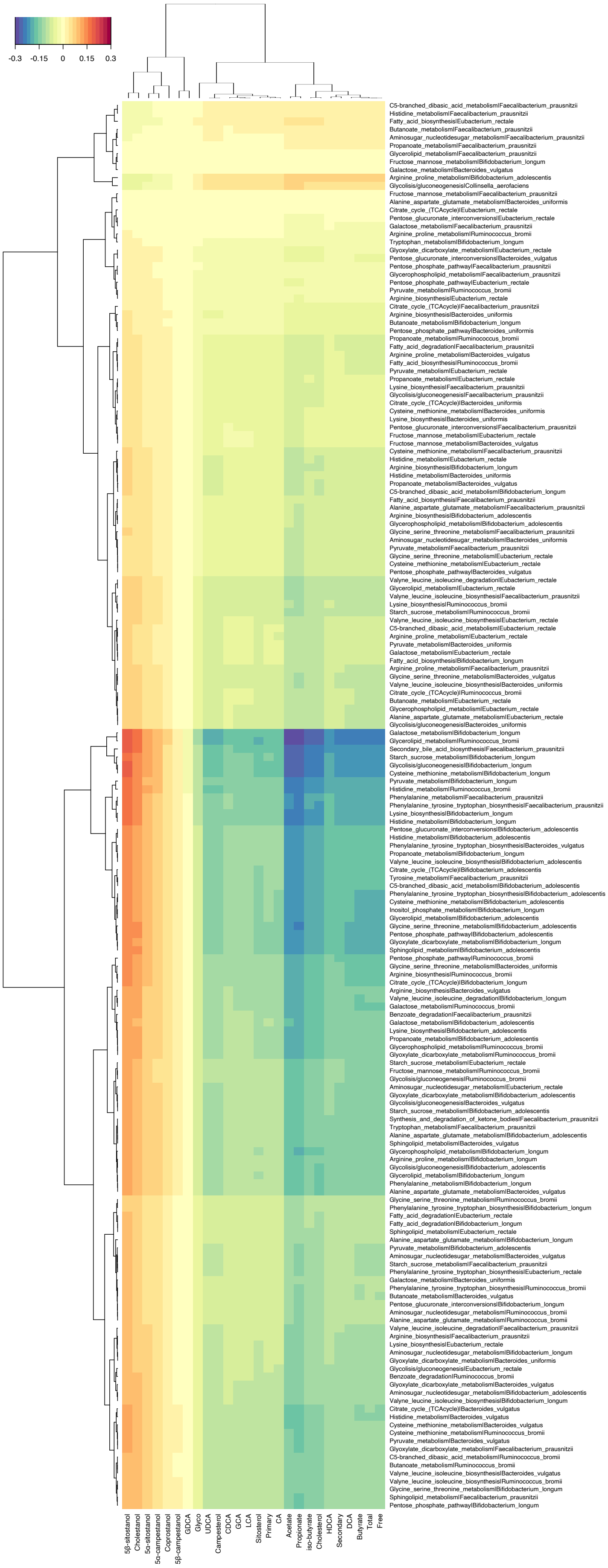

Figure S8. Associations between the transcriptionally active fraction of the gut microbiome and lipidomics measures. Hierarchical clustering obtained with complete linkage method and Pearson correlation as distance, performed on the sparse partial least square (sPLS) regression of transcriptionally active pathways and species of the gut microbiome and host lipids. CA, cholic acid; CDCA, chenodeoxycholic acid; DCA, deoxycholic acid; GCA, glycocholic acid; GDCA, glycodeoxycholic acid; HDCA, hyodeoxycholic acid; LCA, lithocholic acid; UDCA, ursodeoxycholic acid.

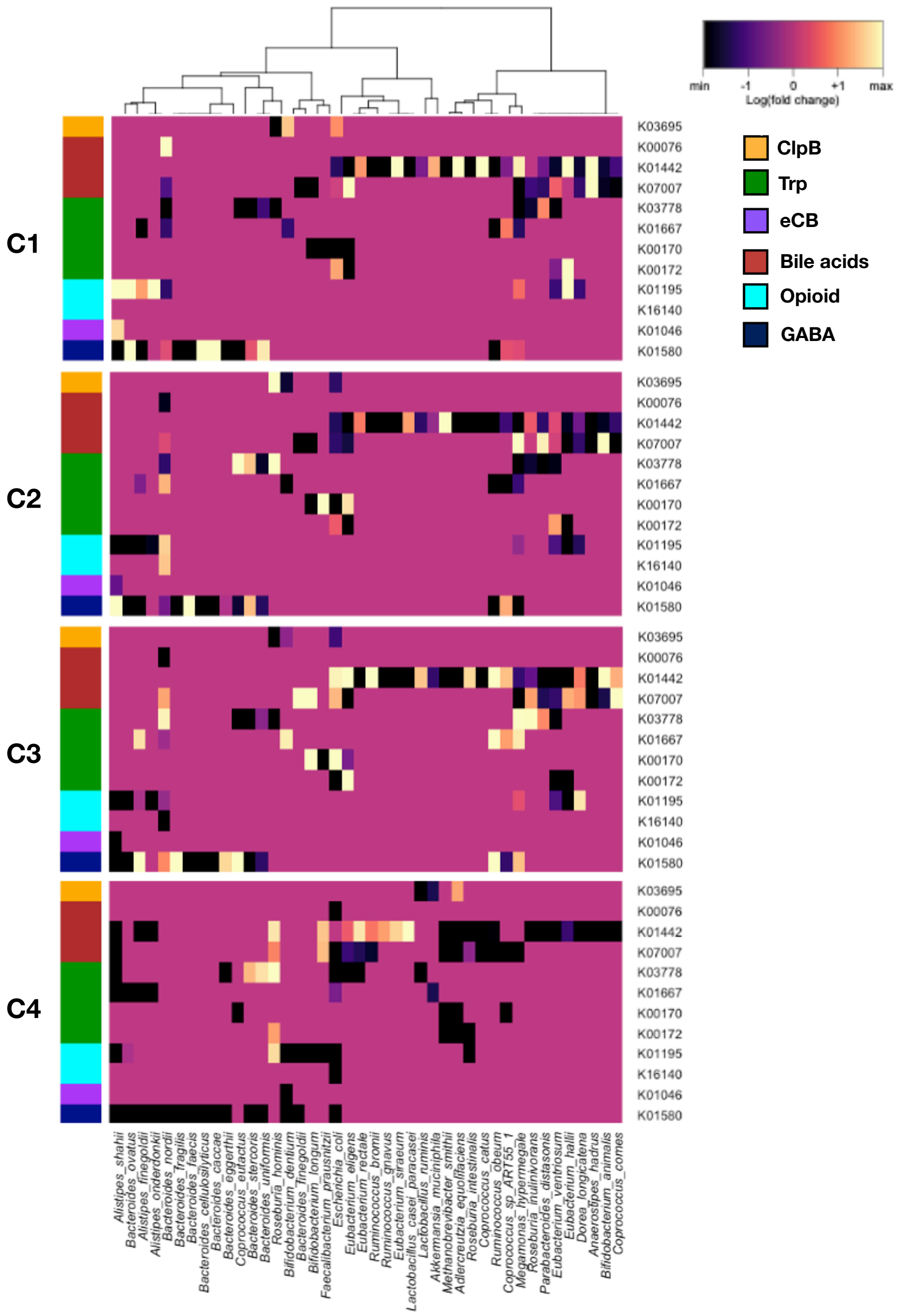

Figure S9. Transcriptional activities of microbiome configurations specifically related to food intake, energy expenditure and neuroendocrine signaling. Average RNA:DNA ratio for each cluster (C1-C4) with respect to the whole cohort, of a selection of key genes involved in ClpB (orange), bile acid (brown), tryptophan (green), opioid (cyan), eCB (violet), and GABA (navy) production, divided by the assigned microbial species. Phylogenetic tree on the top of the heatmap was built using hierarchical Ward linkage clustering based on the Spearman correlation coefficients of the average DNA-normalized transcript abundance profiles for the species with detectable transcriptomes in the whole cohort.

Table S1. Genus-level distribution and differences in the gut microbiota profiles among the four clusters (C1-C4). For each genus, the mean relative abundance and sem (standard error of the mean) are reported, along with Wilcoxon test *p* values (significant values in bold).

	relative abundances								p values					
**Genus**	**mean C1**	**sem C1**	**mean C2**	**sem C2**	**mean C3**	**sem C3**	**mean C4**	**sem C4**	**C1 vs C2**	**C1 vs C3**	**C1 vs C4**	**C2 vs C3**	**C2 vs C4**	**C3 vs C4**
Anaerotruncus	0.0038	0.0013	0.0049	0.0017	0.03	0.0077	0.019	0.0094	0.8	**0.00004**	0.07	**0.0001**	0.1	0.1
Coprobacillus	0.023	0.0094	0.032	0.013	0.048	0.021	0.2	0.11	0.7	0.6	**0.03**	0.4	**0.02**	0.2
Eggerthella	0.026	0.011	0.32	0.081	0.054	0.034	0.13	0.048	**8.00E-06**	0.3	**0.002**	**0.0006**	0.3	**0.03**
Butyricimonas	0.034	0.0076	0.062	0.03	0.14	0.056	0.0054	0.0027	0.08	**0.03**	**0.02**	**0.002**	0.5	**0.0009**
Lachnobacterium	0.035	0.017	0.013	0.0061	0.081	0.058	0.0046	0.0027	**0.03**	0.8	**0.05**	**0.03**	0.7	**0.05**
[Prevotella]	0.057	0.049	0	0	0.13	0.11	0	0	0.07	0.2	0.2	**0.005**	NA	0.06
Slackia	0.059	0.019	0.042	0.021	0.18	0.093	0.16	0.11	0.1	0.1	0.4	**0.007**	0.7	0.09
Desulfovibrio	0.061	0.034	0.022	0.016	0.2	0.081	0.0062	0.0054	0.4	0.1	0.3	**0.03**	0.7	0.06
Veillonella	0.094	0.026	0.3	0.2	0.01	0.0033	0.024	0.014	0.5	**0.0004**	**0.009**	**0.004**	**0.05**	0.7
Adlercreutzia	0.19	0.057	0.074	0.028	0.13	0.033	0.29	0.063	**0.0002**	0.5	**0.04**	**0.005**	**0.0001**	**0.02**
Turicibacter	0.21	0.1	0.16	0.073	0.091	0.026	0.28	0.095	**0.02**	0.5	0.3	0.2	**0.04**	0.08
Catenibacterium	0.26	0.17	0.13	0.13	0.5	0.29	0	0	0.4	0.1	0.1	**0.01**	0.3	**0.01**
Anaerostipes	0.3	0.042	0.19	0.043	0.23	0.057	0.24	0.082	**0.02**	0.1	0.1	0.3	0.3	0.7
Parabacteroides	0.4	0.064	0.6	0.17	0.42	0.066	0.079	0.035	0.2	0.7	**0.0001**	0.1	0.07	**0.00004**
Phascolarctobacterium	0.48	0.1	0.25	0.12	1.06	0.27	0.33	0.22	**0.01**	0.2	**0.05**	**0.002**	0.8	**0.01**
Roseburia	0.62	0.11	0.46	0.099	0.21	0.049	0.24	0.061	0.1	**0.001**	**0.02**	0.4	0.8	0.4
Clostridium	0.67	0.077	0.83	0.14	0.37	0.09	0.25	0.074	0.6	**0.006**	**0.002**	0.04	**0.02**	0.4
[Ruminococcus]	0.99	0.14	2.94	0.59	0.84	0.16	1.37	0.25	**0.001**	0.4	0.1	**0.0008**	0.1	**0.04**
Akkermansia	1.5	0.31	0.39	0.21	6.74	1.71	4.9	1.88	**0.00009**	**0.03**	0.9	**0.00006**	**0.05**	0.3
Lachnospira	1.54	0.36	1.48	0.41	1.13	0.22	0.32	0.14	0.3	0.8	**0.0004**	0.4	**0.04**	**0.002**
Collinsella	1.73	0.21	2.02	0.42	1.95	0.33	4.92	0.91	0.4	0.8	**0.001**	0.5	**0.003**	**0.005**
Coprococcus	1.75	0.3	0.33	0.074	2.38	0.5	0.94	0.31	**3.00E-07**	0.4	0.03	**2.00E-06**	**0.03**	**0.02**
Prevotella	2.23	0.72	0.18	0.12	1.89	0.7	0.083	0.034	**0.002**	0.6	0.08	**0.0002**	0.3	**0.02**
Bifidobacterium	5.55	0.9	11.14	1.38	4.59	1.05	17.84	3.51	**0.001**	0.4	**0.0002**	**0.00008**	**0.05**	**0.0002**
Ruminococcus	5.87	0.46	2.45	0.71	5.36	0.91	6.47	1.11	**1.00E-06**	0.1	0.7	**0.00009**	**0.001**	0.3
Blautia	7.33	0.81	10.68	1.51	3.86	0.61	8.39	1.32	0.1	**0.001**	0.6	**0.00007**	0.5	**0.003**
Bacteroides	8.44	0.98	9.07	1.62	7.68	1.82	2.84	1	0.5	0.2	**0.0004**	0.9	0.09	**0.02**
Faecalibacterium	10.43	0.83	7.61	1.03	5.99	0.97	3.05	0.75	**0.05**	**0.0004**	**6.00E-07**	0.5	**0.03**	**0.04**

Table S2. Food description and average frequency of daily consumption for each microbiota cluster (C1 to C4).

			Microbiota group			
**Food**	**Description**	**Food category**	**C1**	**C2**	**C3**	**C4**
bisquits and sweet snacks	Biscuits, packaged cakes or pastries	Snacks	0.385	0.417	0.215	0.567
cheese	Grated, sliced or spreadable cheese	Cheese	0.25	0.312	0.288	0.333
chocolate	Chocolate candy and bars	Snacks	0.244	0.231	0.182	0.183
eggs	Boiled or fried eggs	Eggs	0.11	0.099	0.092	0.088
fried food	Fried food	Other	0.111	0.077	0.082	0.11
fried potatoes	Fried potatoes	Vegetable	0.084	0.099	0.067	0.076
homemade sandwiches	Hamburger, hotdog, kebab, wrap, falafel	Cereal	0.198	0.149	0.206	0.152
ice cream	Ice cream, milk and fruit based bars	Snacks	0.138	0.135	0.123	0.062
meat	Fresh and fried meat	Meat Products	0.088	0.117	0.086	0.138
milk and yoghurt	Milk and plain sweetened yoghurt	Dairy Products	0.481	0.664	0.464	0.683
not homemade sandwiches	Hamburger, hotdog, kebab, wrap, falafel	Cereal	0.076	0.064	0.092	0.112
olive oil	Vegetable dressing	Fat and oils	0.658	0.784	0.561	0.776
pizza	Pizza	Cereal	0.146	0.14	0.185	0.133
sausages	Cold cuts and preserved meat product	Meat Products	0.038	0.072	0.029	0.043
seasonings and condiments	Butter, margarine on bread, pesto, ragù	Condiments	0.214	0.244	0.089	0.217
slide ham	Cold cuts and preserved meat product	Meat Products	0.116	0.139	0.12	0.167
sweet and salty snacks	Packaged cakes or savoury pastries and fritters	Snacks	0.204	0.244	0.155	0.16
sweetened drinks	Sweetened soft beverages	Drinks	0.118	0.195	0.092	0.1
wine	Alcoholic beverages	Drinks	0.189	0.172	0.273	0.16

Table S3. Summary of dietary groups and microbiota clusters for each of the 100 enrolled women. Women were stratified based on addictive eating behavior into 4 groups: NW_NA (normal-weight, non-addicted), O_LA (overweight/obese, low-addicted), O_HA (overweight/obese, high-addicted), and O_DHA (overweight/obese, high-addicted with food addiction diagnosis). For microbiota clusters and dietary groups, see Fig. 3 and Fig. 5, respectively.

SampleID	Group	Microbiota cluster	Diet group
NEU10B	O_LA	C3	D1
NEU116B	O_HA	C2	D3
NEU117B	O_NA	C4	D3
NEU118B	O_NA	C2	D1
NEU119B	O_LA	C2	D1
NEU11B	O_NA	C4	D1
NEU127B	O_LA	C4	D3
NEU129B	O_NA	C2	D2
NEU12B	O_LA	C2	D1
NEU134B	O_HA	C3	D2
NEU139B	O_NA	C1	D3
NEU13B	O_LA	C1	D3
NEU144B	O_NA	C3	D2
NEU14B	O_LA	C4	D1
NEU152B	O_NA	C1	D1
NEU153B	O_LA	C4	D1
NEU155B	O_LA	C3	D3
NEU160B	O_NA	C2	D1
NEU161B	O_LA	C1	D1
NEU163B	O_LA	C4	D1
NEU164B	O_LA	C2	D3
NEU167B	O_NA	C3	D2
NEU16B	O_HA	C2	D1
NEU170B	O_NA	C1	D2
NEU172B	O_NA	C2	D1
NEU174B	O_HA	C2	D2
NEU176B	O_NA	C1	D2
NEU177B	O_LA	C3	D3
NEU178B	O_HA	C3	D1
NEU179B	O_LA	C1	D1
NEU17B	O_HA	C2	D1
NEU180B	O_LA	C1	D2
NEU18B	O_HA	C1	D1
NEU19B	O_HA	C3	D1
NEU1B	O_NA	C3	D3
NEU20B	O_HA	C3	D3
NEU21B	O_HA	C4	D1
NEU22B	O_HA	C2	D1
NEU23B	O_HA	C2	D1
NEU25B	O_NA	C1	D1
NEU26B	O_NA	C1	D1
NEU27B	O_LA	C3	D3
NEU28B	O_NA	C1	D3
NEU29B	O_NA	C2	D1
NEU2B	O_NA	C2	D1
NEU30B	O_NA	C1	D1
NEU31B	O_LA	C1	D3
NEU32B	O_HA	C3	D2
NEU33B	O_HA	C3	D3
NEU34B	O_NA	C2	D3
NEU35B	O_LA	C2	D3
NEU36B	O_NA	C2	D2
NEU37B	O_NA	C2	D1
NEU38B	O_NA	C2	D1
NEU39B	O_LA	C2	D3
NEU3B	O_LA	C1	D3
NEU4B	O_LA	C4	D1
NEU5B	O_NA	C2	D1
NEU6B	O_NA	C2	D1
NEU7B	O_NA	C3	D3
NEU8B	O_HA	C1	D1
NEU9B	O_LA	C2	D1

SampleID	Group	Microbiota cluster	Diet group
NC120B	NW_NA	C1	D1
NC121B	NW_NA	C2	D2
NC122B	NW_NA	C1	D3
NC123B	NW_NA	C1	D3
NC124B	NW_NA	C2	D3
NC125B	NW_NA	C2	D3
NC126B	NW_NA	C1	D1
NC128B	NW_NA	C2	D1
NC130B	NW_NA	C2	D1
NC131B	NW_NA	C1	D1
NC132B	NW_NA	C1	D3
NC133B	NW_NA	C3	D3
NC135B	NW_NA	C2	D1
NC136B	NW_NA	C1	D1
NC137B	NW_NA	C4	D2
NC138B	NW_NA	C4	D1
NC140B	NW_NA	C1	D3
NC141B	NW_NA	C3	D2
NC142B	NW_NA	C1	D2
NC143B	NW_NA	C4	D2
NC145B	NW_NA	C1	D2
NC146B	NW_NA	C3	D3
NC147B	NW_NA	C1	D1
NC148B	NW_NA	C2	D2
NC149B	NW_NA	C4	D1
NC150B	NW_NA	C3	D1
NC151B	NW_NA	C1	D2
NC154B	NW_NA	C4	D1
NC156B	O_HA	C4	D3
NC157B	NW_NA	C3	D1
NC159B	NW_NA	C1	D2
NC162B	NW_NA	C3	D1
NC165B	NW_NA	C2	D2
NC166B	NW_NA	C1	D1
NC168B	NW_NA	C3	D2
NC171B	NW_NA	C1	D1
NC173B	NW_NA	C3	D2
NC175B	NW_NA	C2	D1

Table S4. Loading scores obtained from sPLS correlation analysis between metatranscriptomics and lipidomics datasets. UDCA, ursodeoxycholic acid; HDCA, hyodeoxycholic acid; GCA, glycocholic acid; CA, cholic acid; CDCA, chenodeoxycholic acid; GDCA, glycodeoxycholic acid; DCA, deoxycholic acid; LCA, lithocholic acid; Free, free bile acids; Glyco, glycine-conjugated bile acids; Primary, primary bile acids; Secondary, secondary bile acids; Total, total bile acids. See also Fig. 9.

	comp1	comp2
Alanine_aspartate_glutamate_metabolism|*Bacteroides_uniformis*	0.009235	0.04616256
Alanine_aspartate_glutamate_metabolism|*Bacteroides_vulgatus*	-0.0520611	-0.0977455
Alanine_aspartate_glutamate_metabolism|*Bifidobacterium_adolescentis*	0.08556606	-0.0572281
Alanine_aspartate_glutamate_metabolism|*Bifidobacterium_longum*	0.15288117	0.01184153
Alanine_aspartate_glutamate_metabolism|*Eubacterium_rectale*	-0.0137712	-0.0794428
Alanine_aspartate_glutamate_metabolism|*Faecalibacterium_prausnitzii*	0.14664802	0.05194782
Alanine_aspartate_glutamate_metabolism|*Ruminococcus_bromii*	-0.0391005	-0.0701839
Aminosugar_nucleotidesugar_metabolism|*Bacteroides_uniformis*	-0.0166901	-0.0101881
Aminosugar_nucleotidesugar_metabolism|*Bacteroides_vulgatus*	-0.0359378	-0.0556378
Aminosugar_nucleotidesugar_metabolism|*Bifidobacterium_adolescentis*	0.03401977	0.06051082
Aminosugar_nucleotidesugar_metabolism|*Bifidobacterium_longum*	0.16476177	-0.0168718
Aminosugar_nucleotidesugar_metabolism|*Eubacterium_rectale*	-0.0095053	-0.1217103
Aminosugar_nucleotidesugar_metabolism|*Faecalibacterium_prausnitzii*	0.02032995	0.04422082
Aminosugar_nucleotidesugar_metabolism|*Ruminococcus_bromii*	-0.0430462	-0.06318
Arginine_biosynthesis|*Bacteroides_uniformis*	0.17587216	0.01734575
Arginine_biosynthesis|*Bacteroides_vulgatus*	-0.04415	-0.0850094
Arginine_biosynthesis|*Bifidobacterium_adolescentis*	0.17454724	0.01654483
Arginine_biosynthesis|*Bifidobacterium_longum*	0.17555978	0.00256697
Arginine_biosynthesis|*Eubacterium_rectale*	0.19039125	0.02537051
Arginine_biosynthesis|*Faecalibacterium_prausnitzii*	0.15123445	-0.016064
Arginine_biosynthesis|*Ruminococcus_bromii*	-0.0451281	-0.1084412
Arginine_proline_metabolism|*Bacteroides_vulgatus*	-0.0532033	-0.112923
Arginine_proline_metabolism|*Bifidobacterium_adolescentis*	0.10478334	0.22925757
Arginine_proline_metabolism|*Bifidobacterium_longum*	-0.0367454	-0.0869794
Arginine_proline_metabolism|*Eubacterium_rectale*	0.1014597	-0.0562661
Arginine_proline_metabolism|*Faecalibacterium_prausnitzii*	0.05237896	-0.0650532
Arginine_proline_metabolism|*Ruminococcus_bromii*	0.00157108	0.02449597
Benzoate_degradation|*Faecalibacterium_prausnitzii*	-0.0118867	-0.1170003
Benzoate_degradation|*Ruminococcus_bromii*	-0.0390719	-0.0685037
Butanoate_metabolism|*Bacteroides_vulgatus*	-0.0382804	-0.0681517
Butanoate_metabolism|*Bifidobacterium_longum*	-0.0038901	-0.0139872
Butanoate_metabolism|*Eubacterium_rectale*	0.03207697	-0.0690874
Butanoate_metabolism|*Faecalibacterium_prausnitzii*	-0.0369	-0.0111378
Butanoate_metabolism|*Ruminococcus_bromii*	-0.0273715	-0.0793332
C5-branched_dibasic_acid_metabolism|*Bifidobacterium_adolescentis*	-0.0445968	-0.0990282
C5-branched_dibasic_acid_metabolism|*Bifidobacterium_longum*	0.16868302	0.02599513
C5-branched_dibasic_acid_metabolism|*Eubacterium_rectale*	0.02757989	-0.0595254
C5-branched_dibasic_acid_metabolism|*Faecalibacterium_prausnitzii*	-0.0048205	0.00776443
C5-branched_dibasic_acid_metabolism|*Ruminococcus_bromii*	-0.0268406	-0.0795628
Citrate_cycle_(TCAcycle)|*Bacteroides_uniformis*	-0.0123036	0.00459165
Citrate_cycle_(TCAcycle)|*Bacteroides_vulgatus*	-0.0365719	-0.0724681
Citrate_cycle_(TCAcycle)|*Bifidobacterium_adolescentis*	-0.0473634	-0.0979925
Citrate_cycle_(TCAcycle)|*Bifidobacterium_longum*	-0.0393426	-0.0976341
Citrate_cycle_(TCAcycle)|*Eubacterium_rectale*	0.18700245	0.1251242
Citrate_cycle_(TCAcycle)|*Faecalibacterium_prausnitzii*	-0.0329655	-0.0110352
Citrate_cycle_(TCAcycle)|*Ruminococcus_bromii*	-0.0384286	-0.0482036
Cysteine_methionine_metabolism|*Bacteroides_uniformis*	-0.0534738	-0.1084937
Cysteine_methionine_metabolism|*Bacteroides_vulgatus*	-0.0391717	-0.0734806
Cysteine_methionine_metabolism|*Bifidobacterium_adolescentis*	0.02683465	-0.0895375
Cysteine_methionine_metabolism|*Bifidobacterium_longum*	-0.0732872	-0.1672791
Cysteine_methionine_metabolism|*Eubacterium_rectale*	0.16930047	-0.011449
Cysteine_methionine_metabolism|*Faecalibacterium_prausnitzii*	-0.0574743	-0.0478324
Cysteine_methionine_metabolism|*Ruminococcus_bromii*	-0.0459755	-0.0823733
Fatty_acid_biosynthesis|*Bifidobacterium_longum*	-0.054355	-0.1331028
Fatty_acid_biosynthesis|*Eubacterium_rectale*	0.01180928	0.0446595
Fatty_acid_biosynthesis|*Faecalibacterium_prausnitzii*	0.13161096	0.01468158
Fatty_acid_biosynthesis|*Ruminococcus_bromii*	0.17714228	0.00963077
Fatty_acid_degradation|*Bifidobacterium_longum*	-0.0333133	-0.0450722
Fatty_acid_degradation|*Eubacterium_rectale*	-0.0518408	-0.1097163
Fatty_acid_degradation|*Faecalibacterium_prausnitzii*	0.0090592	0.01122093
Fructose_mannose_metabolism|*Bacteroides_vulgatus*	0.02085311	0.06321989
Fructose_mannose_metabolism|*Bifidobacterium_longum*	0.07990683	0.15969279
Fructose_mannose_metabolism|*Eubacterium_rectale*	-0.0370269	-0.0419938
Fructose_mannose_metabolism|*Faecalibacterium_prausnitzii*	-0.0282621	-0.0004705
Fructose_mannose_metabolism|*Ruminococcus_bromii*	-0.036474	-0.0801744
Galactose_metabolism|*Bacteroides_uniformis*	-0.047041	-0.0751224
Galactose_metabolism|*Bacteroides_vulgatus*	0.03692171	0.10931652
Galactose_metabolism|*Bifidobacterium_adolescentis*	0.00647719	0.00436452
Galactose_metabolism|*Bifidobacterium_longum*	0.03833331	-0.1000509
Galactose_metabolism|*Eubacterium_rectale*	-0.0263877	-0.0431515
Galactose_metabolism|*Faecalibacterium_prausnitzii*	0.09279556	0.07212299
Galactose_metabolism|*Ruminococcus_bromii*	0.01570023	-0.1021153
Glycerolipid_metabolism|*Bifidobacterium_adolescentis*	-0.0480968	-0.1046292
Glycerolipid_metabolism|*Bifidobacterium_longum*	-0.0410826	-0.0778977
Glycerolipid_metabolism|*Eubacterium_rectale*	-0.0512837	-0.1034357
Glycerolipid_metabolism|*Faecalibacterium_prausnitzii*	-0.0179159	-0.0428639
Glycerolipid_metabolism|*Ruminococcus_bromii*	-0.074291	-0.1840143
Glycerophospholipid_metabolism|*Bifidobacterium_adolescentis*	0.00107726	0.01120388
Glycerophospholipid_metabolism|*Bifidobacterium_longum*	-0.0322393	-0.0694025
Glycerophospholipid_metabolism|*Eubacterium_rectale*	0.02161453	-0.0434535
Glycerophospholipid_metabolism|*Faecalibacterium_prausnitzii*	0.17490803	0.01309418
Glycerophospholipid_metabolism|*Ruminococcus_bromii*	-0.0339757	-0.0988365
Glycine_serine_threonine_metabolism|*Bacteroides_uniformis*	-0.0518845	-0.0806993
Glycine_serine_threonine_metabolism|*Bacteroides_vulgatus*	-0.0321992	-0.0480549
Glycine_serine_threonine_metabolism|*Bifidobacterium_adolescentis*	-0.0472385	-0.1053129
Glycine_serine_threonine_metabolism|*Bifidobacterium_longum*	0.02529456	-0.069869
Glycine_serine_threonine_metabolism|*Eubacterium_rectale*	0.01854273	0.03127098
Glycine_serine_threonine_metabolism|*Faecalibacterium_prausnitzii*	-0.0498555	-0.0392114
Glycine_serine_threonine_metabolism|*Ruminococcus_bromii*	0.15703109	-0.0227147
Glycolisis/gluconeogenesis|*Bacteroides_uniformis*	0.09945992	-0.0004881
Glycolisis/gluconeogenesis|*Bacteroides_vulgatus*	-0.0354176	-0.0764224
Glycolisis/gluconeogenesis|*Bifidobacterium_adolescentis*	0.09829039	-0.017426
Glycolisis/gluconeogenesis|*Bifidobacterium_longum*	0.04242188	-0.0863507
Glycolisis/gluconeogenesis|*Collinsella_aerofaciens*	0.03319151	0.10454868
Glycolisis/gluconeogenesis|*Eubacterium_rectale*	0.00479905	-0.0763129
Glycolisis/gluconeogenesis|*Faecalibacterium_prausnitzii*	0.11917693	-0.030391
Glycolisis/gluconeogenesis|*Ruminococcus_bromii*	0.12179793	-0.0460131
Glyoxylate_dicarboxylate_metabolism|*Bacteroides_uniformis*	-0.065396	-0.0942943
Glyoxylate_dicarboxylate_metabolism|*Bacteroides_vulgatus*	-0.0359098	-0.0637778
Glyoxylate_dicarboxylate_metabolism|*Bifidobacterium_adolescentis*	0.12345606	-0.036593
Glyoxylate_dicarboxylate_metabolism|*Bifidobacterium_longum*	-0.0561564	-0.1537441

	comp1	comp2
Glyoxylate_dicarboxylate_metabolism|*Eubacterium_rectale*	-0.0018626	-0.0259434
Glyoxylate_dicarboxylate_metabolism|*Faecalibacterium_prausnitzii*	0.15205624	-0.0005026
Glyoxylate_dicarboxylate_metabolism|*Ruminococcus_bromii*	0.03103988	-0.0607227
Histidine_metabolism|*Bacteroides_uniformis*	-0.0371093	-0.05323
Histidine_metabolism|*Bacteroides_vulgatus*	-0.0304953	-0.0840892
Histidine_metabolism|*Bifidobacterium_adolescentis*	-0.0312191	-0.048457
Histidine_metabolism|*Bifidobacterium_longum*	-0.0598143	-0.1302051
Histidine_metabolism|*Eubacterium_rectale*	-0.0504765	-0.0841557
Histidine_metabolism|*Faecalibacterium_prausnitzii*	-0.0202314	0.0205642
Histidine_metabolism|*Ruminococcus_bromii*	0.0425901	-0.0804564
Inositol_phosphate_metabolism|*Bifidobacterium_longum*	-0.0183562	-0.109768
Lysine_biosynthesis|*Bacteroides_uniformis*	-0.048137	-0.1029858
Lysine_biosynthesis|*Bifidobacterium_adolescentis*	0.00215933	0.01680654
Lysine_biosynthesis|*Bifidobacterium_longum*	-0.0760526	-0.1552683
Lysine_biosynthesis|*Eubacterium_rectale*	0.02126343	-0.0921346
Lysine_biosynthesis|Faecalibacterium_prausnitzii	0.13341229	-0.0173083
Lysine_biosynthesis|*Ruminococcus_bromii*	0.12420193	-0.0249484
Pentose_glucuronate_+E19	-0.0069456	-0.0172927
Pentose_glucuronate_interconversions|*Bifidobacterium_adolescentis*	0.00663459	-0.0593316
Pentose_glucuronate_interconversions|*Bifidobacterium_longum*	-0.0051937	-0.0319584
Pentose_glucuronate_interconversions|*Eubacterium_rectale*	0.20427309	0.06553809
Pentose_glucuronate_interconversions|*Faecalibacterium_prausnitzii*	0.14343727	0.05493857
Pentose_phosphate_pathway|*Bacteroides_uniformis*	0.00811933	0.04530702
Pentose_phosphate_pathway|*Bacteroides_vulgatus*	-0.0045012	0.0111527
Pentose_phosphate_pathway|*Bifidobacterium_adolescentis*	-0.0484244	-0.0878174
Pentose_phosphate_pathway|*Bifidobacterium_longum*	0.12240484	-0.074938
Pentose_phosphate_pathway|*Eubacterium_rectale*	0.18759885	0.02499292
Pentose_phosphate_pathway|*Faecalibacterium_prausnitzii*	0.15597607	0.03684897
Pentose_phosphate_pathway|*Ruminococcus_bromii*	-0.0220488	-0.111932
Phenylalanine_metabolism|*Bifidobacterium_longum*	-0.0540729	-0.1256542
Phenylalanine_metabolism|*Faecalibacterium_prausnitzii*	-0.0271721	-0.1023913
Phenylalanine_tyrosine_tryptophan_biosynthesis|*Bacteroides_vulgatus*	-0.0619938	-0.1662977
Phenylalanine_tyrosine_tryptophan_biosynthesis|*Bifidobacterium_adolescentis*	-0.0503946	-0.1011115
Phenylalanine_tyrosine_tryptophan_biosynthesis|*Bifidobacterium_longum*	-0.0264878	-0.0627147
Phenylalanine_tyrosine_tryptophan_biosynthesis|*Eubacterium_rectale*	-0.0459295	-0.0911387
Phenylalanine_tyrosine_tryptophan_biosynthesis|*Faecalibacterium_prausnitzii*	-0.0767918	-0.1693213
Phenylalanine_tyrosine_tryptophan_biosynthesis|*Ruminococcus_bromii*	-0.0358508	-0.0531028
Propanoate_metabolism|*Bacteroides_vulgatus*	-0.0292632	-0.0406072
Propanoate_metabolism|*Bifidobacterium_adolescentis*	0.09747076	-0.0349509
Propanoate_metabolism|*Bifidobacterium_longum*	-0.0218885	-0.0670602
Propanoate_metabolism|*Eubacterium_rectale*	-0.0213239	-0.0245049
Propanoate_metabolism|*Faecalibacterium_prausnitzii*	-0.002103	-0.0306922
Propanoate_metabolism|*Ruminococcus_bromii*	-0.0121883	0.01073714
Pyruvate_metabolism|*Bacteroides_uniformis*	-0.0432859	-0.0641322
Pyruvate_metabolism|*Bacteroides_vulgatus*	-0.0364955	-0.0702486
Pyruvate_metabolism|*Bifidobacterium_adolescentis*	0.15290735	0.00404538
Pyruvate_metabolism|*Bifidobacterium_longum*	-0.0224948	-0.1010304
Pyruvate_metabolism|*Eubacterium_rectale*	-0.0518731	-0.1182136
Pyruvate_metabolism|*Faecalibacterium_prausnitzii*	0.03191536	0.08941692
Pyruvate_metabolism|*Ruminococcus_bromii*	-0.0313049	0.01081402
Secondary_bile_acid_biosynthesis|*Faecalibacterium_prausnitzii*	-0.0708559	-0.1899872
Sphingolipid_metabolism|*Bacteroides_vulgatus*	-0.0707435	-0.1558627
Sphingolipid_metabolism|*Bifidobacterium_adolescentis*	-0.0480328	-0.1020751
Sphingolipid_metabolism|*Eubacterium_rectale*	0.00302496	0.00268982
Sphingolipid_metabolism|*Faecalibacterium_prausnitzii*	0.02724378	-0.0687131
Starch_sucrose_metabolism|*Bifidobacterium_adolescentis*	0.10931292	0.02872667
Starch_sucrose_metabolism|*Bifidobacterium_longum*	0.05843321	-0.097843
Starch_sucrose_metabolism|*Eubacterium_rectale*	0.04243033	-0.0692128
Starch_sucrose_metabolism|*Faecalibacterium_prausnitzii*	0.12795212	-0.0511098
Starch_sucrose_metabolism|*Ruminococcus_bromii*	-0.0032485	-0.0397679
Synthesis_and_degradation_of_ketone_bodies|*Faecalibacterium_prausnitzii*	-0.0397837	-0.1345995
Tryptophan_metabolism|*Bifidobacterium_longum*	0.05427486	0.12993408
Tryptophan_metabolism|*Faecalibacterium_prausnitzii*	-0.0432389	-0.1350475
Tyrosine_metabolism|*Faecalibacterium_prausnitzii*	-0.0647138	-0.1234953
Valyne_leucine_isoleucine_biosynthesis|*Bacteroides_uniformis*	-0.0369219	-0.0414568
Valyne_leucine_isoleucine_biosynthesis|*Bacteroides_vulgatus*	-0.0172274	-0.0506811
Valyne_leucine_isoleucine_biosynthesis|*Bifidobacterium_adolescentis*	-0.0191291	-0.0752678
Valyne_leucine_isoleucine_biosynthesis|*Bifidobacterium_longum*	0.12890713	-0.0390076
Valyne_leucine_isoleucine_biosynthesis|*Eubacterium_rectale*	0.02421914	-0.0658155
Valyne_leucine_isoleucine_biosynthesis|*Faecalibacterium_prausnitzii*	-0.0273044	-0.070694
Valyne_leucine_isoleucine_biosynthesis|*Ruminococcus_bromii*	-0.0281565	-0.0877048
Valyne_leucine_isoleucine_degradation|*Bifidobacterium_longum*	-0.0533973	-0.1627613
Valyne_leucine_isoleucine_degradation|*Eubacterium_rectale*	-0.0543576	-0.1103114
Valyne_leucine_isoleucine_degradation|*Faecalibacterium_prausnitzii*	-0.0004455	-0.1226968
UDCA	0.0318571	0.18728833
HDCA	0.12381823	0.22497026
GCA	0.26273764	0.16915474
CA	0.09803688	0.17612961
CDCA	0.09461606	0.166439
GDCA	0.63568879	0.01496889
DCA	0.07808028	0.23783547
LCA	0.13910779	0.16952029
Free	0.09889973	0.24130769
Glyco	0.49128508	0.1079967
Primary	0.10273992	0.17421449
Secondary	0.09290263	0.2314309
Total	0.10274423	0.24103386
Cholesterol	-0.045817	0.26048194
Coprostanol	0.04587252	-0.057621
Cholestanol	-0.0064043	-0.1737653
Sitosterol	-0.0015629	0.17930497
5α-sitostanol	0.02353579	-0.1898743
5β-sitostanol	0.09345976	-0.1293214
Campesterol	-0.034006	0.18740311
5α-campestanol	-0.0353578	-0.0182533
5β-campestanol	0.06144568	-0.1049988
Acetate	0.25021637	0.28911942
Propionate	0.28235266	0.29704989
Butyrate	0.12100257	0.24159476
Iso-butyrate	0.09453227	0.25803138

Table S5. Summary of the main distinctive features of the four microbiota clusters in terms of clinical, dietary and lipidomic profiles. BITE, Bulimic Investigatory Test Edinburgh; NDT, non-distinctive tracts; SCFAs, short-chain fatty acids.

	microbiota profile			
	**C1**	**C2**	**C3**	**C4**
**clinical characteristics**	NDT	↑ uric acid, BITE severity score	NDT	↑BITE symptoms score<br>↓ uric acid
**diet**	↑ fiber intake	↑ energy intake<br>↓ fiber, fat intake	NDT	↑ energy, carbohydrate intake<br>↓ fat intake
**lipidomics**	↑ SCFAs	↑ SCFAs, cholesterol, bile acids	↑ converted sterols<br>↓ SCFAs	↑bile acids<br>↓ SCFAs
